# Cloning, Expression, Sequence Analysis and Homology Modeling of the Prolyl Endoprotease from *Eurygaster integriceps* Puton

**DOI:** 10.3390/insects5040762

**Published:** 2014-10-22

**Authors:** Ravi Chandra Yandamuri, Ranjeeta Gautam, Charles Darkoh, Vanitha Dareddy, Mustapha El-Bouhssini, Beatrice A. Clack

**Affiliations:** 1 Department of Biology and Biotechnology, Stephen F. Austin State University, P.O. Box 13003, SFA Station, Nacogdoches, TX 75962, USA; E-Mails: yendamuri.ravi@gmail.com (R.C.Y.); ranjeeta_gautam2000@yahoo.com (R.G.); vanithareddy006@yahoo.com (V.D.); 2 University of Texas Health Science Center, School of Public Health, Center for Infectious Diseases, 1200 Hermann Pressler Dr., RAS E715, Houston, TX 77030, USA; E-Mail: cdarkoh@hotmail.com; 3 Biodiversity and Integrated Gene Management Program, International Center for Agricultural Research in the Dry Areas (ICARDA), Rabat Office P.O. Box 6299 Rabat-Instituts, Rabat, Morocco; E-Mail: M.BOHSSINI@CGIAR.ORG

**Keywords:** Hemiptera, Scutelleridae, prolyl endoprotease, gluten, serine protease, cDNA, homology modeling, insect, wheat

## Abstract

*Eurygaster integriceps* Puton, commonly known as sunn pest, is a major pest of wheat in Northern Africa, the Middle East and Eastern Europe. This insect injects a prolyl endoprotease into the wheat, destroying the gluten. The purpose of this study was to clone the full length cDNA of the sunn pest prolyl endoprotease (spPEP) for expression in *E. coli* and to compare the amino acid sequence of the enzyme to other known PEPs in both phylogeny and potential tertiary structure. Sequence analysis shows that the 5ꞌ UTR contains several putative transcription factor binding sites for transcription factors known to be expressed in *Drosophila* that might be useful targets for inhibition of the enzyme. The spPEP was first identified as a prolyl endoprotease by Darkoh *et al.*, 2010. The enzyme is a unique serine protease of the S9A family by way of its substrate recognition of the gluten proteins, which are greater than 30 kD in size. At 51% maximum identity to known PEPs, homology modeling using SWISS-MODEL, the porcine brain PEP (PDB: 2XWD) was selected in the database of known PEP structures, resulting in a predicted tertiary structure 99% identical to the porcine brain PEP structure. A Km for the recombinant spPEP was determined to be 210 ± 53 µM for the zGly-Pro-pNA substrate in 0.025 M ethanolamine, pH 8.5, containing 0.1 M NaCl at 37 °C with a turnover rate of 172 ± 47 µM Gly-Pro-pNA/s/µM of enzyme.

## 1. Introduction

*Eurygaster integriceps* Puton, commonly known as sunn pest, belongs to the order Hemiptera and family Scutelleridae. This true bug is considered a major pest of wheat crops in Northern Africa, the Middle East and Eastern Europe. In addition to direct reduction in wheat crops during the life cycle of the bug, the sunn pest also injects saliva containing hydrolytic and proteolytic enzymes into the grains during feeding. The mechanism of injection is most likely similar to that shown for a closely related bug, brown marmorated stink bug, *Halyomorpha halys* (Hemiptera: Pentatomidae), which secretes its proteases in the watery saliva through a stylet in the beak [1]. The salivary glutenase, characterized as a prolyl endoprotease (spPEP) by Darkoh *et al.* [2], assists in penetration and pre-oral digestion of the grain contents by degrading the high molecular weight gluten proteins of the wheat. The spPEP remains in the grain after the bugs have finished feeding and continue to cause extensive damage to the gluten proteins when the grain is milled and used as dough. The degradation of the gluten causes bread made from such dough to be weak, sticky and to have reduced volume and an unusually heavy texture [3,4]. The dough does not rise in the oven and often burns. As little as 2%–5% sunn pest-contaminated grains render the whole lot unacceptable for baking purposes [5,6]. Darkoh *et al.* [2] showed the spPEP to be a serine protease through inhibition by phenylmethylsulfonyl fluoride (PMSF) and, specifically, a prolyl endoprotease, which cleaved peptide bonds at the carboxyl terminal side of the GlyPro-pNA substrate. The enzyme was partially purified by Darkoh *et al.* [2], exhibiting a Km of 65.3 ± 1.8 µM for the GlyPro-pNA substrate at pH 8, 22 °C, with maximum activity between pH 8–10 and 25 °C–35 °C in 25 mM ethanolamine buffer. The turnover number could not be determined by Darkoh, because the enzyme was not purified to homogeneity.

A recombinant form of the enzyme responsible for gluten degradation is required to determine environmentally safe inhibitors to reduce crop losses. The spPEP is unique, because its natural substrates are large gluten proteins; Perez *et al.* [7] showed the enzyme to preferentially hydrolyze high molecular weight glutenins that are as large as 140 kD. Every [8] and Darkoh [2] showed a general specificity for whole gluten containing both the high and low molecular weight glutenins and gliadins, as well as specificity for the dipeptide GPpNA.

The primary focus of this study was to clone the full transcript of the spPEP and produce an active recombinant prolyl endoprotease from the sunn pest. Prior to submission of the nucleic acid sequence obtained from this study, the only nucleotide sequence reported for true bugs in the National Center for Biotechnology Information (NCBI) was amylase for *Eurygaster integriceps*; therefore, degenerate primers were designed based on known insect PEP sequences to probe cDNA from each of the life stages of *Eurygaster integriceps*. The cDNA from three different life stages of the sunn pest was constructed with only the adult sunn pest expressing the mRNA for the spPEP. The 5ꞌ and 3ꞌ untranslated regions of the transcript were analyzed for possible transcription factor binding sites and secondary structure motifs that might be used for translational control for future studies to determine mechanisms of inhibition of the enzyme through either a biological pesticide or the use of transgenic wheat. Inhibitors must be specific to the sunn pest due to human consumption of the wheat; therefore, knowing the complete sequence of the spPEP transcript and comparing it to others, including that of mammals, is important for specifically targeting the spPEP. A phylogenetic comparison of the spPEP amino acid sequences to other known PEPs places the spPEP on a branch with *Daphnia pulex* separate from other known insects and several nodes distant from mammalian PEPs. Structure analysis is also important for inhibitor design. Despite being at most only 56% identical to all of the known PEP sequences, homology modeling of the resulting amino acid sequence shows the spPEP to be highly homologous in its tertiary structure to that of the porcine brain PEP, which recognizes only short peptides less than 30 amino acids long.

## 2. Experimental Section

### 2.1. Harvesting Sunn Pest of Various Life Stages

Sunn pest nymphs and actively feeding adults were harvested from the experimental wheat fields of the International Center for Agricultural Research in the Dry Areas (ICARDA), Syria, during the months of April and June, 2010, respectively. The overwintering adults were collected from a small forest near the wheat fields of the ICARDA experimental station in fall 2010. Approximately two hundred insects of each life stage were collected. In order to increase the mRNA levels that encode for the insect salivary enzymes, especially PEP, actively feeding insects were starved for 24 h and then allowed to feed on wheat grains in plastic containers, with aeration and light provided.

### 2.2. Synthesis of cDNA

#### 2.2.1. Total RNA Isolation

The actively feeding insects were powdered in liquid nitrogen using a mortar and pestle. Total RNA was isolated from ten to fifteen insects of each life stage using the SV total RNA isolation system from Promega Corp. (Madison, WI, USA) according to the manufacturer’s instructions.

#### 2.2.2. mRNA Enrichment

In order to ensure contaminating bacterial mRNA was not amplified in downstream PCR reactions, eukaryotic mRNA was isolated from the total RNA using the PolyATract mRNA isolation system (Promega Corp., Madison, WI, USA).

#### 2.2.3. First Strand cDNA Synthesis

The purified mRNA from the three different life stages was converted to first strand cDNA using the SMARTer RACE cDNA synthesis kit (Clonetech Inc., Mountain View, CA, USA). Primers, buffers and all enzymes were included in the kit. For each of the life stages, two reaction buffer mixes, one for 5ꞌ RACE-ready cDNA and one for 3ꞌ RACE-ready cDNA, were prepared according to the manufacturer’s instructions using between 3.5 and 7 ng of mRNA for the 5ꞌ RACE and 3ꞌ RACE ready cDNA.

### 2.3. Amplification of spPEP Transcript

#### 2.3.1. Design of Degenerate Primers

Degenerate primers shown in Table 1 were designed using COnsensus-DEgenerate Hybrid Oligonucleotide Primer (CODEHOP) [9] with the consensus sequence obtained from the ClustalW2 alignment [10] of the following insect PEP amino acid sequences: *Drosophila melanogaster* (NP_610129), *Culex quinquefasciatus* (XP_001843671), *Aedes aegypti* (XP_001659779), *Nasonia vitripennis* (XP_001603578) and *Apis mellifera* (XP_395364).

**Table 1:** Gene-specific and degenerate primers designed from the known sequence information and the deduced sequence information during the time course of the project. Primer 3 software [11], available online, was used to design the following gene-specific primers, and the CODEHOP program [9], available online, was used for designing the degenerate primers. All of the primers were purchased from Sigma-Aldrich. Each primer had a region of conserved degeneracy at the 3ꞌ end (lower case) and a non-degenerate 5ꞌ clamp (upper case).

Primer Name	Sequence 5ꞌ→3ꞌ
P1gen	CCCCTACAGGTGGCTGgargayccnga
P24gen	TGAACTTGTGGAACCTCAGCatrtccatnac
P1	CCCCTACAGGTGGCTGGA
265	TGGTCGTCATCGATTTTGAA
415	TTCACAGTTTGGTGGATGGA
707	CCTGGAACAAAACGGAAAAA
PEP Start	ATGAAAAAGTTCCAATACCCTGAAGCTCGG
1747	CGCTTCTGCAAACATAAGGGGAGGA
312	ACGTCACCCAATTTTCTTCG
434	TCCATCCACCAAACTGTGAA
980	TCGCCAGACAACTCTTATTG
Lic PEP Start	TATTTTCAATCCTACGTAATGAAAAAGTTCCAATACCCTG
Lic PEP Stop	CCCTCAATATTATACGGGTCA AATGATCTGACAAAC

#### 2.3.2. Amplification from Degenerate Primers

**Figure 1:**
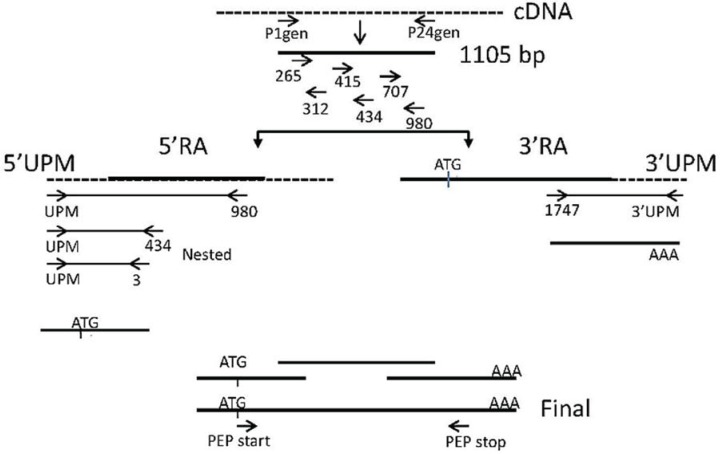
PCR reactions performed to amplify the PEP cDNA. The known and deduced sequence regions are represented by solid lines. The unknown sequence regions are represented by dotted lines. The arrows represent the direction of amplification from the gene-specific primers that successfully amplified the unknown regions of the PEP gene. The universal primer mix (UPM) contained short and long universal primers that bound to their complementary adaptors that were placed on the 5ꞌ end during 5ꞌ RACE and on the 3ꞌ end for 3ꞌ RACE first strand cDNA synthesis from mRNA.

Polymerase chain reaction (PCR) was performed with KlenTaq LA polymerase (DNA Polymerase Technology, Inc. St. Louis, MO, USA) as illustrated in Figure 1 using the 5ꞌ and 3ꞌ RACE-ready cDNA, described in Section 2.2.3, as the template. Degenerate primer sequences P1gen and P24gen (Table 1) were used. PCR conditions consisted of an initial denaturation at 95 °C for 4 min followed by 32 cycles of 95 °C for 45 s, 50 °C for 45 s and 72 °C for 3 min with a final extension at 72 °C for 5 min.

#### 2.3.3. Amplification of the 5ꞌ and 3ꞌ Ends

The 5ꞌ RACE ready cDNA contained the SMARTer IIA oligo on their 5ꞌ end, making the 5ꞌ RACE cDNA complimentary to the universal primers (UPM) provided in the SMARTer RACE kit. Based on the sequence information of the P1gen-P24gen amplicon (1,105 bp), internal gene-specific primers (GSP) (Table 1) were designed to walk through the cDNA towards both the 5ꞌ and 3ꞌ ends as the sequence became known, as illustrated in Figure 1. The GSP Primer design in this case was performed using the Primer 3 software, available online [11]. The parameters for the PCR were an initial denaturation at 95 °C for 4 min; 30 cycles consisting of a 1-min melt at 95 °C, a 1-min annealing at the Tm for the specific primer pair and a 1-min/kb extension at 68 °C; and finally, a 5-min final extension at 68 °C.

#### 2.3.4. Confirmation of cDNA Clones

The amplicons were gel purified using the Gel Purification system from Promega Corp. (Madison, WI, USA) and cloned into the pGEM-T Easy vector (Promega Corp., Madison, WI, USA.) using the pGEM-T Easy vector System II Protocol for ligation and transformation. Colonies were selected for *EcoR1* digestion to confirm that the insert was present, and then, positive colonies were sent for sequencing (Amplicon Express Inc., Pullman, WA, USA).

#### 2.3.5. Final Assembly of the PEP cDNA Sequence

The raw sequences were vector trimmed to remove vector contamination using either NEB cutter 3.0 or DNA Baser (DNA Baser Sequence Assembler v3.x (Heracle BioSoft S.R.L.). A final contig of all fragment sequences was obtained, which included the 5ꞌ untranslated region (5ꞌ UTR), the complete open reading frame (ORF) and the 3ꞌ untranslated region (3ꞌ UTR). The resultant contig was submitted to BLASTX [12] at the NCBI web-based portal to confirm that the sequence was indeed similar to other insect PEP sequences.

### 2.4. Cloning of the Sunn Pest PEP Construct into the Expression Vector

Primers with the start ATG (primer: PEPstart) and stop (primer: PEPstop) codons were used to amplify the ORF from the PEP cDNA, which was subsequently cloned into the pGEM-T EASY vector (Promega Corp., Madison, WI, USA), as described before. Following the confirmation of the amplified ORF as PEP, the gene was ligated into the pNYCOMPS-LIC-ccdB-FH10T+ (N term) vector, a bacterial expression vector purchased from Arizona State University (ASU) Biodesign Institute. The vector includes an inducible T7 promoter, an *N* terminal flag epitope and a 10X His tag. In addition, it has a Kanamycin resistance gene and allows for ligation-independent cloning (LIC) and IPTG induction. The complete PEP gene was amplified by PCR with the LIC-modified PEP Start (Lic PEP start) and PEP Stop (Lic PEP stop) primers (Table 1) using the PEP clone from Section 2.3.5 as the template; the amplicon was designated as LIC-PEP. The vector was linearized with *Sna*BI (NEB, Ipswich, MA, USA) and subsequently treated with T4 DNA polymerase in the presence of 10 mM dGTP according to the ASU protocol [13] for making LIC ends. Similarly, the amplified LIC-PEP DNA was also treated with T4 DNA polymerase (Promega Corp., Madison, WI, USA) in the presence of only dCTPs at the same concentration as the dGTP above. For each reaction, 2 µL of vector were combined with 4 µL of LIC-PEP. The components were allowed to anneal at room temperature for 60 min. Following incubation, 2 µL of 25 mM EDTA was added to each reaction tube and incubated at room temperature for 5 min. Two microliters of each LIC reaction were transformed into the BL21(DE3)-competent *E. coli* cells (NEB, Ipswich, UK).

### 2.5. Sequence Analysis

The 5ꞌ UTR sequence was submitted to TFFSearch [14] to determine the consensus sequences present for transcription factor binding. The coding sequence, which started at base pair 330 of the complete cDNA and ended at 2,483 bp, was submitted to BLASTX at the NCBI portal using the eukaryote, insect, fungi, bacteria and Archaea databases. A ClustalW2 [10] alignment of the sunn pest PEP was performed with the top 25 hits obtained from the BLASTX (Figure S2), as well as with the PEP sequences from the six known insects used to generate the consensus primers used for cDNA synthesis, as well as four PEP sequences, whose structure has been determined, the porcine PEP sequence and three bacterial PEP sequences to determine the degree of identity and homology. Additionally, the sequence was scanned for putative signal sequences using SignalP 4.1 available as a web portal [15].

ClustalW2 was used to align the top 25 sequences obtained from the BLASTX. A phylogenetic tree was constructed using Phylogeny.fr [16] with the ClustalW2 alignment file as input. Homology modeling was performed using the web-based SWISS-MODEL [17,18], which searches known structures for the best fit. USCF Chimera 1.8.1 [19] was then used for visualization. The Chimera 1.8.1 MatchMaker tool was used to plot the spPEP predicted tertiary structure aligned with the reference structure of the porcine brain PEP (2XDW.pdb). Using the Alignment assessment tool, structural differences between the spPEP sequence and the porcine sequence were identified.

### 2.6. Expression and Purification of Recombinant PEP

#### 2.6.1. Expression

Multiple colonies that contained the full-length PEP in the LIC vector were grown on a small scale to determine the highest expressing clones. Ten milliliters of culture in LB plus Kanamycin were grown to an OD_600_ of 0.4, at which time, IPTG was added to a final concentration of 0.4 mM. The cultures continued to grow overnight at 37 °C. The cells were harvested by centrifugation at 12,000 rpm for 10 min. The cell pellets were resuspended in 500 µL of disruption buffer as described by Vora *et al.* [20] to obtain the soluble fraction of protein with the following modifications: 0.1 M sodium phosphate, pH 7.5, was used in lieu of Tris, and DTT was omitted. These substitutions were made to keep the protease inactive throughout purification. The cells were lysed using 200 µg/mL lysozyme and incubated for 30 min on ice with occasional stirring. DNAse and RNase (Sigma-Aldrich, St. Louis, MO, USA) were added to a final concentration of 5 µg/mL. A short pulse of sonication was performed to further disrupt the cells. The samples were centrifuged after lysis at maximum rpm in a microcentrifuge for 10 min to separate the soluble protein from the inclusion bodies possibly containing insoluble PEP. The pellet was resuspended in 500 µL of disruption buffer plus 7 M urea [20] to obtain the solubilized inclusion body proteins. A Bradford assay was performed on each of the soluble and inclusion body fractions. Laemmli sample buffer was added to the 50 µg of protein, heated to 95 °C for 3 min and loaded on to a 10% SDS PAGE gel, which was run at 100 V, until the dye front migrated just off the gel. The gel was stained with Coomassie Blue R250.

After identification of the highest expressing clones, the culture was scaled up to 6 L in a New Brunswick BioFlo110 Fermenter. The culture was grown at 37 °C with 200 rpm agitation until an OD_600_ of 0.6 was reached. IPTG was then added to a final concentration of 0.4 mM, at which time the culture temperature was shifted to 28 °C for overnight growth. The culture was centrifuged at 7,000× *g* for 30 min, after which the supernatant was discarded. The pellet was lysed using CellLytic Express (Sigma-Aldrich) resuspended in 0.1 M sodium phosphate, pH 7.5, according to the manufacturer’s instructions, to obtain the soluble fraction of expressed protein. No protease inhibitors were added to the buffer, due to unknown effects on the spPEP in downstream experiments. The remaining pellet representing the insoluble protein was resuspended in 7 M urea in disruption buffer according to Vora *et al.* [20].

#### 2.6.2. Affinity Purification of PEP

Both the soluble and the insoluble spPEP were purified in a single step using Ni affinity chromatography. The enzyme had to be eluted in a buffer that would inhibit the activity of the enzyme; otherwise, the enzyme proteolyzed itself and disappeared. As previously shown by Darkoh *et al.* [2], the enzyme is active at pH 6–10; therefore, the pH could not be adjusted to stop the enzyme activity. Active enzyme requires 0.1 M NaCl and 1 mM DTT; therefore, these components were omitted until the enzyme activity was assessed. The soluble fraction was batch adsorbed to 10 mL of PerfectPro Ni-NTA agarose (5-Prime, Inc., Gaithersburg, MD, USA) equilibrated in 0.1 M sodium phosphate buffer, pH 7.5 (Buffer A). The column was washed with Buffer A until the A_260_ returned to zero. The protein was eluted using a shallow gradient of imidazole from 0 M to 0.5 M over a total volume of 500 mL. The insoluble protein was resuspended in disruption buffer containing 7 M urea and bound to 10 mL of Ni resin by batch adsorption. The column was washed using Buffer A containing 7 M urea. The insoluble protein was refolded on the column using a gradient of decreasing urea (7 M to 0 M over 100 mL at 2 mL/min). The enzyme was then eluted with a gradient of increasing imidazole from 0 mM imidazole to 0.5 M imidazole in 100 mL of 0.1 M sodium phosphate, pH 7.5. Samples exhibiting enzyme activity as evidenced of zGPpNA hydrolysis were pooled, concentrated and the imidazole removed using Centriprep filter cartridges with a 50 kD cut-off (Millipore, Billerica, MA, USA).

### 2.7. Measurement of PEP Activity

#### 2.7.1. zGly-Pro-pNA Assay for PEP Activity

Each of the protein lysates, from the soluble fraction and from the refolded insoluble fraction, as well as the purified enzyme, were assayed for PEP activity using zGly-Pro-pNA (benzyloxycarbonyl-Gly-Pro-p-nitroanilide) (Bachem Americas, Inc., Torrance, CA, USA). The reaction buffer consisted of 0.025 M ethanolamine, pH 8.5, with 0.3 mM zGly-Pro-pNA substrate. In a 96-well microtiter plate, 150 µL of reaction buffer were added to all wells. Enough enzyme in 0.1 M sodium phosphate, pH 7.5, was added to separate wells, so that 50 µL of enzyme could be added to each well containing substrate to start the reaction. Immediately after the addition of the enzyme, the plate was placed in a Benchmark Plus microtiter plate reader (Bio-Rad Laboratories, Hercules, CA, USA). The absorbance at 410 nm was measured in kinetic mode. The ε_410_ for cleaved zGPpNA is 8,800 L·mol^−1^·cm^−1^ [2]. One unit of activity is defined as the amount of enzyme that produces a change in absorbance of 0.1 at 410 nm per minute [2]. The specific activity of the PEP in each fraction is reported as µM of product·min^−1^.

#### 2.7.2. Glutenase Activity

The glutenase assay, developed by Every [8], was used to determine the glutenase activity of the PEP. In this assay, 200 µL of freshly prepared 5% w/v wheat gluten (obtained at local grocery store) in 0.1 M ethanolamine with the addition of 0.1 M NaCl and 1 mM DTT, pH 8.5, were incubated with 50 µL of PEP. Gluten, in buffer without enzyme, was used as a blank. Samples were incubated in a 37 °C water bath for 2 h with vigorous shaking every 30 min. After incubation, 10% freshly prepared SDS was added to a final concentration of 2% and incubated further for 30 min with vigorous shaking every 5 min. The gel height was measured after centrifugation at 3,000× *g* for 10 min. A 1-mm change in height corresponded to one unit of enzyme [8].

#### 2.7.3. Kinetic Assay

Protein concentration was determined using the Bradford assay (Bio-Rad, Hercules, CA) in the case of the spPEP. To determine the Km and turnover number of the recombinant enzyme for the zGPpNA substrate, the substrate concentration was varied from 0 mM to 0.3 mM. The reaction buffer was the same as described above in Section 2.7.1. Purified recombinant spPEP at a concentration of 0.65 µg/µL (8.125 µM) was added per well. The A_410_ was measured every 15 s for 20 min. The initial velocity was plotted as a function of substrate concentration using Excel. A double reciprocal plot (1/[S] *vs.* 1/v) was used to determine the Km and Vmax. The turnover number for the enzyme was determined by dividing the Vmax by the µM of PEP using a molecular weight of 80,000, as previously determined by Darkoh *et al.* [2].

#### 2.7.4. Gluten Isolation and Digestion

Gluten, purchased from the grocery store, was extracted according to van den Broeck *et al.* [21] using 50% isopropanol in 0.1 M sodium phosphate plus 1 mM DTT, pH 7.5. The protein concentration was determined using the Modified Lowry Assay (Thermo Scientific, IL). The digestion with spPEP consisted of incubating 2 mg/mL of gluten fraction with 65 µg of spPEP in PBS plus 1 mM DTT in 300 µL at 37 °C over time up to 90 min. Samples were periodically mixed by vortexing followed by removal of 25 µL every 15 min for SDS PAGE analysis of the digestion. The reaction was stopped by putting each sample into 5 µL of 6× Laemmli sample buffer containing 6 M urea and heating to 95 °C for 3 min. Each sample was applied to a 4%–20% SDS PAGE mini-gel (Bio-Rad, Hercules, CA) for separation of the proteins. The gels were stained with SyproRuby (Bio-Rad, Hercules, CA) according to the manufacturer’s instructions and imaged using a Typhoon Trio Plus (GE-Lifesciences, Pittsburgh, PA, USA) with the excitation laser set at 532 nm and the emission laser set at 610 nm.

## 3. Results and Discussion

### 3.1. Total RNA Isolation and mRNA Enrichment

Total RNA was successfully obtained from all samples. Actively feeding adults resulted in the highest yield of mRNA at 1.88 ng/µL with an A_260_/A_280_ ratio of 2.1. mRNA from each of the life stages was used as a template to generate the RACE-ready cDNA.

### 3.2. SMARTer RACE cDNA Synthesis

The enriched mRNA for each life stage was converted to first strand cDNA resulting in 5ꞌ and 3ꞌ RACE-ready cDNA with concentrations ranging from 62 ng/µL to 77 ng/µL and purity (A_260_/A_280_) from 1.6 for the nymphs, 1.8 for the over-wintering adults and 2.0 for the actively feeding adults (first year).

### 3.3. Amplification Using Degenerate Primers

PCR amplification of the actively feeding adult cDNA was the only cDNA that produced amplicons. Therefore, subsequent amplifications used only the cDNA from the actively feeding adults. A 1,105-base pair fragment returned a match for prolyl endopeptidase from different organisms, including the insects, whose sequences were used to generate the degenerate primers (Section 2.3.1). This 1,105-bp sequence was used to design the GSPs that were used in subsequent amplifications, as illustrated in Figure 1 and listed in Table 1. The primer names were designated according to the base pair position in the 1,105-bp PCR amplicon.

### 3.4. Rapid Amplification of cDNA Ends (RACE)

As illustrated in Figure 1 for both the 5ꞌ direction and 3ꞌ direction, the gene specific primers, 265, 415 and 707 (Table 1), were paired with the universal primer mix for 3ꞌ RACE (3ꞌ RACE-ready cDNA as the template). Gene-specific primers, 312, 434 and 980 (Table 1), were paired with the universal primer mix for 5ꞌ RACE (5ꞌ RACE-ready cDNA as template). No distinct band was observed in any of the amplification reactions; however, faint smearing was observed [22]. Aliquots of these reactions were used as templates for nested PCR.

### 3.5. Sequence Analysis

The obtained sequences were vector trimmed using DNA Baser and aligned with all resulting sequences to obtain the final transcript. The deduced sequence included the start methionine at 348 bp resulting in 347 bp of the 5ꞌ untranslated region (the UTR is shown in the solid box in Figure S1), the stop codon at 2,483 bp and a 3ꞌ UTR region followed by the poly-A tail of 29 adenine residues. No signal sequence was identified using the web-based software, SignalP 4.1 [15]. Combining the 5ꞌ contig and the resulting 3ꞌ contig, the complete cDNA was found to be 2,822 bp in length, as shown in Figure S1. The open reading frame was deposited into GenBank and is listed as Accession Number EU934738.3. The 5ꞌ UTR consisted of several conserved transcription factor binding sites, as shown in Figure S2 and listed in Table S1. All of the transcription factors that bind to the sites identified [23,24,25,26,27,28] have been well studied in *Drosophila* and correspond to factors produced in the saliva glands consistent with the spPEP having regulated expression in the salivary glands of the sunn pest.

**Figure 2:**
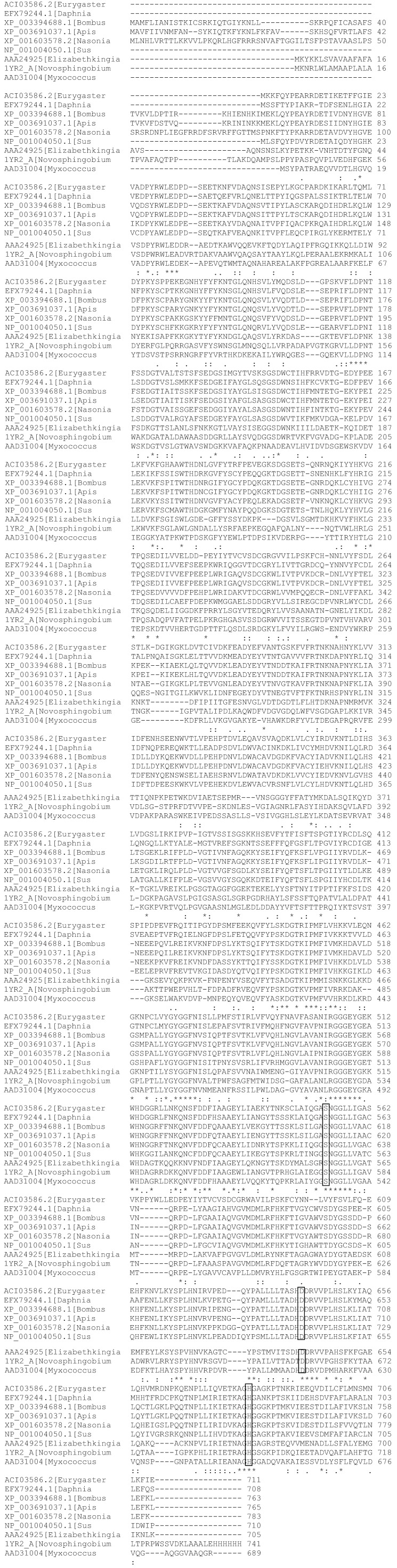
Sunn pest PEP amino acid sequence aligned with PEP sequences of *Daphnia pulex* (gb|EFX79244.1), *Nasonia vitripennis* (XP_001603578.2), *Bombus terrestris* (XP_003394688.1), *Apis florea* (XP_003691037.1), *Sus scrofa* (NP_001004050.1), *Sphingomonas capsulate* (1YR2_A), *Elizabethkingia meningoseptica* (AAA24925) and *Myxococcus xanthus* (AAD31004). ClustalW [10] was used to generate the alignment. The amino acids comprising the catalytic triad are boxed. * Identical residues, conserved residues.

The deduced amino acid sequence aligned with the insect PEP amino acid sequences used to create the degenerate primers, bacterial sequences for which the structure has been determined and that of *Sus susex* is shown in Figure 2. The first twenty hits from BLASTX [12] are shown in the Supplemental Data as Table S3. The first 19 hits were PEPs of closely-related insects with 85% coverage. The highest identity was found to be 56% to *Bombus terrestris* and *Daphnia pulex*. *Nasonia vitripennis* and *Apis mellifera* were both only 55% identical, and *Camponotus floridanus* and *Harpegnathos saltator* were 53% identical*.* Further supporting the fact that the sequenced DNA is a prolyl endopeptidase, the spPEP sequence contained the conserved domain for serine proteases of type S9A with the conserved catalytic domain consisting of Ser-Asp-His aligning (boxed residues in Figure 2). A phylogenetic tree was constructed using the alignment and was performed using Phylogeny.fr [16] obtained from ClustalW2 with the top 25 hits from BLASTX in addition to the sequences of the insects used in the degenerate primer design (if they did not come up in the BLASTX result), as well as four species for which the three-dimensional structure has been determined, the three bacterial sequences (*Sphingomonas capsulate*, Eliz*abethkingia meningoseptica* and *Myxococcus xanthus*) and the porcine PEP (Figure S3). The spPEP sequence separated into a node shared with *Daphnia pulex* with both the spPEP and the *Daphnia* PEP being separated by several nodes from the other insect PEPs and even further removed from the mammalian PEPs. The bacterial PEPs were at most 39% identical, as determined from BLASTX (Table S4).

### 3.6. Structure Analysis of spPEP Based on Amino Acid Sequence

**Figure 3:**
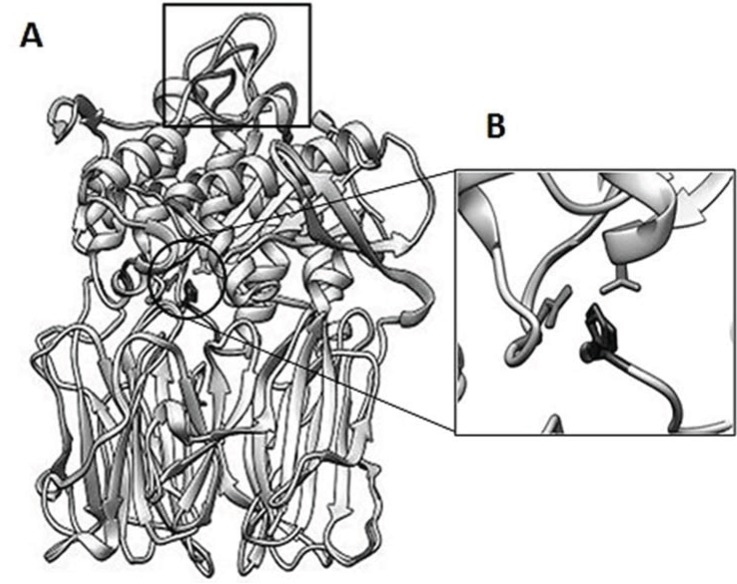
Homology model of the spPEP. SWISS-MODEL was used to fit the spPEP to a best fit to porcine PEP (2XDW.pdb). The Chimera 1.8.1 Match Align tool was used to generate the aligned structure (**A**). Differences between the structures are shown in solid black, which were identified using the structure assessment tool in Chimera. The boxed portion of the structure shows the highest deviation. The catalytic core residues are circled and align in close proximity to each other. The catalytic core residues are shown in an expanded view (**B**).

The amino acid sequence was submitted to homology modeling using SWISS-MODEL [17,18]. The modeling consisted of comparing the submitted sequence to the available structures in the PDB database. The structure for the porcine brain PEP (PDB: 2XDW) with an inhibitor peptide bound was selected to be the most conserved sequence and structure despite being distantly removed from the spPEP phylogenetically. As shown in Figure 3, the spPEP aligned 99% to that of the porcine brain PEP. A PDB file was generated from SWISS-MODEL, which was then, visualized using Chimera 1.8.1 [19]. The largest deviations from the porcine structure occurred in the loop regions, in particular residues 626–627 of the spPEP (black) compared to residues 632–636 of the porcine PEP (grey) and residues 567–578 of the spPEP (black) compared to residues 570–681 of the porcine structure. Other minor deviations were identified using the alignment assessment tool in Chimera; these are indicated in black, as well. The conserved catalytic residues (spPEP/porcine), Ser553/554, Asp642/641 and His681/680, align at the same location in both structures (Figure 3B). Despite having only 50% identity in amino acid sequence, the modeling suggests that the structure is highly conserved, questioning why and how the spPEP recognizes large and small substrates compared to other PEPs in this family, which, to date, have only been shown to recognize substrates less than 3 kD in size.

### 3.7. Cloning and Expression of Active spPEP

**Figure 4:**
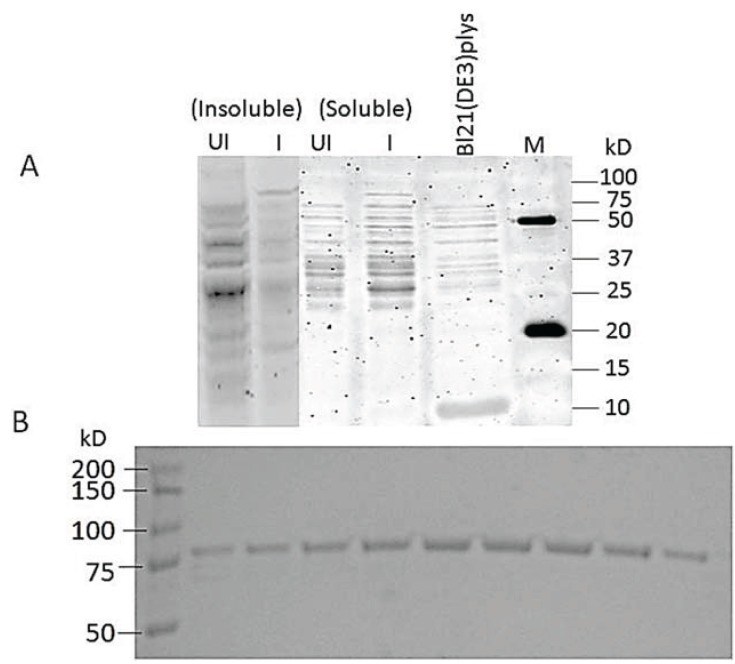
(**A**) SDS polyacrylamide gel of the cell lysates of uninduced and IPTG-induced Bl21(DE3)pLysS cultures expressing spPEP. UI = uninduced, I = induced with 0.4 mM IPTG. The cell lysate of the host without the recombinant enzyme construct is shown for comparison. There was no insoluble fraction for the host alone for comparison. (**B**) SDS PAGE (10%) of recombinant spPEP eluted from a Ni-NTA agarose column. Protein was stained with Coomassie Blue R250.

An amplicon of 2,153 bp was obtained and successfully ligated into the LIC vector. Figure 4A shows a SDS PAGE of the cell lysate of the soluble and insoluble fractions. Significant amounts of recombinant PEP were found in the soluble fraction of the induced cells, but there was also PEP found in the insoluble fraction (Figure 4A). Figure 4B shows the Coomassie-stained 80-kD spPEP eluted from the nickel column on a 10% SDS PAGE gel. A total of 18 mg, approximately 95% pure, was obtained from the soluble fraction of 6 L of culture (Figure 4B).

#### 3.7.1. z-Gly-Pro-pNA Assay for PEP Activity

**Figure 5:**
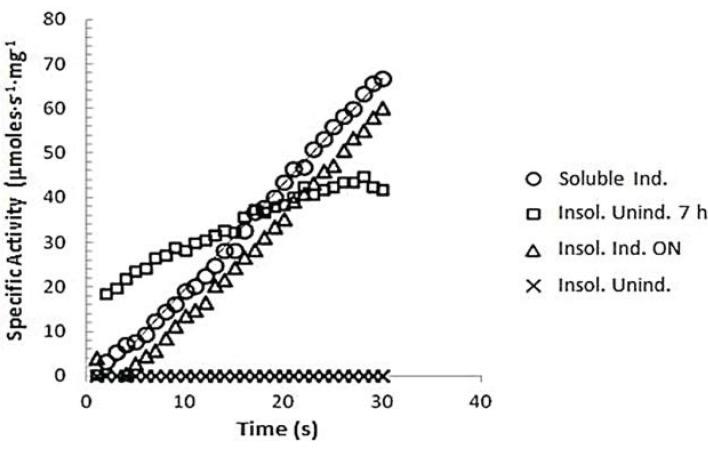
GPpNA assay of recombinant spPEP. The buffer was exchanged with 50 mM Tris, pH 8.0, containing 0.1 M NaCl for both the induced and uninduced soluble and inclusion body lysates. The reactions were started by the addition of 100 µL of 3 mM GPpNA to 100 µL of lysate. The A_410_ was converted to specific activity (µM·s^−1^·mg^−1^ of total protein) for the respective sample using a ε_410_ = 8,800 L·mol^−1^·cm^−1^.

After the buffer exchange of PEP into the 25 mM ethanolamine buffer, pH 8.5, containing 0.1M NaCl and 1 mM DTT, the PEP enzyme activity was measured in both uninduced and induced protein fractions. The enzyme activity for both the soluble crude extract and the refolded insoluble protein fraction are shown in Figure 5. Both the soluble extract and the refolded insoluble protein fraction exhibited PEP specific activity. The specific activity was approximately the same for the induced samples whether soluble or refolded insoluble at 2.4 µM·s^−1^·mg^−1^ of total protein. Within the 9-min assay, the substrate was not depleted for these samples. Enzyme activity was observed in the uninduced refolded insoluble protein as a result of leaky expression; however, the activity was reduced within about 2.5 min, reaching only 2/3 of the activity of the induced samples. Very little spPEP was observed in this fraction at 80 kD on the SDS PAGE (Figure 4A), which would be the size of the full-length enzyme. No protease inhibitors were added to the cell lysates in order to keep the spPEP from being inhibited, so it is possible that the small amount of activity observed in this fraction might have been due to unstable fragments of the enzyme.

#### 3.7.2. Determination of Km of spPEP

**Figure 6:**
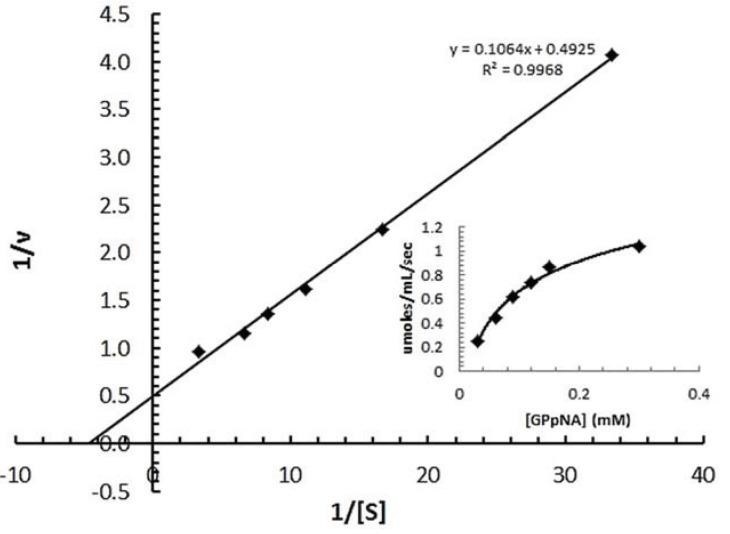
Michaelis-Menten kinetics of the recombinant spPEP from the soluble protein fraction. PGpNA concentrations were varied from 0 to 300 µM in 0.1 M ethanolamine buffer, pH 8.5, containing 0.1 M NaCl and 1 mM DTT. The reaction was started by the addition 130 µg of purified spPEP per well. The A_410_ was monitored over time. The Km and Vmax were determined from the double reciprocal plot, 1/v vs 1/[GPpNA]. The inset shows the initial velocities obtained for each substrate concentration plotted *vs.* substrate concentration to generate the Michaelis-Menten curve. A Vmax of 106 µmole/mL/min was used with the µM concentration of PEP to determine the turnover number.

The spPEP purified from the soluble fraction exhibited a Km similar to that determined by Darkoh [2] for the enzyme purified from infested wheat. A representative Michaelis–Menten kinetics is shown in Figure 6 using zGPpNA as the substrate. The inset shows the v *vs.* [S] plot. The enzyme Km for the peptide substrate was determined to be 211 ± 53 µM in 25 mM ethanolamine, pH 8.5, 0.1 M NaCl, 1 mM DTT. Darkoh *et al.* [2] reported a Km of 65.3 µM for the enzyme purified from the infested wheat and analyzed in 25 mM ethanolamine, pH 8.5. One primary reason for the difference in Km could be the level of purity of the enzyme. The enzyme in Darkoh’s study was only partially purified, whereas the recombinant protein was greater than 95% pure, as determined from the SDS PAGE (Figure 4B). The presence of contaminating proteins might actually help in the stability and binding of the substrate to the PEP. Additional studies need to be performed, such as the addition of BSA, to the reaction to test this hypothesis. Because the enzyme was pure, the turnover number for the spPEP was able to be determined. The turnover number was 172 ± 47 µM of peptide/s/µmole of spPEP.

#### 3.7.3. Gluten Assay

**Figure 7:**
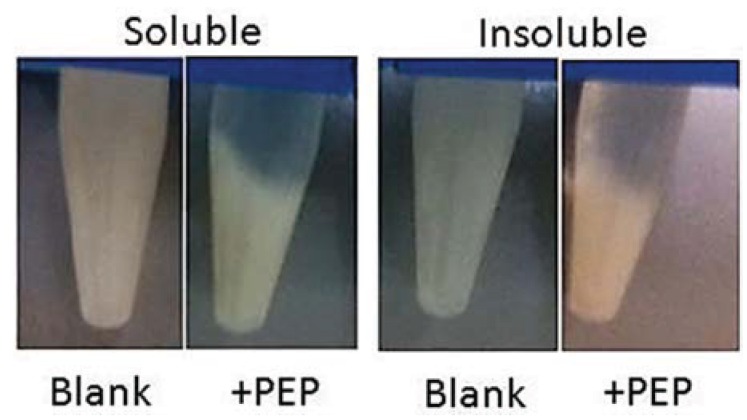
Glutenase assay of recombinant spPEP in both soluble and inclusion body lysates. Buffer was exchanged to 0.1 M ethanolamine, 0.1 M NaCl, pH 8.5. Reaction conditions were at 37 °C, for 2 h. One unit of enzyme activity is defined as a 1-mm change in gel height [8].

**Figure 8:**
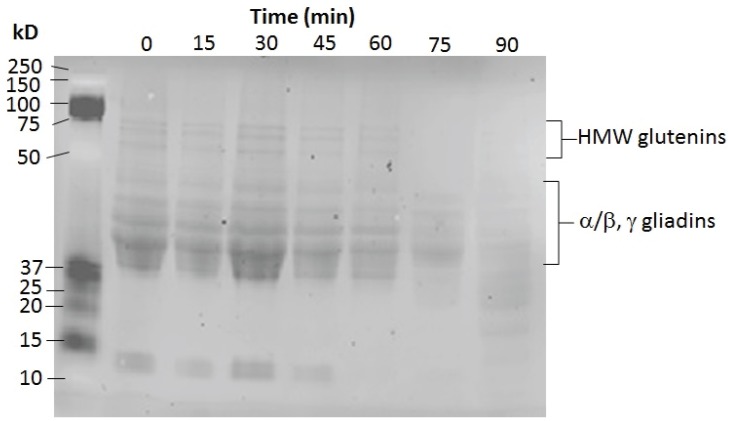
SDS PAGE analysis of the digestion of total gluten by spPEP. spPEP (60 µg) was incubated with 400 µg of total gluten for a total of 90 min at 37 °C. Every 15 min, a volume of the reaction that would contain 50 µg of gluten was removed, added to 6× Laemmli sample buffer, heated and loaded onto the gel. The molecular weight marker was the Kaleidoscope marker from Bio-Rad Laboratories.

Both the soluble fraction and the refolded insoluble fraction of the induced lysates exhibited glutenase activity. As shown in Figure 7, very similar glutenase activities were observed for the enzyme in the soluble protein lysate at 0.58 units and the enzyme in the refolded insoluble protein at 0.63 units. Direct analysis of the peptide products from the glutenase assay was not possible due to the fragments being too small for gel electrophoresis. Therefore, a separate reaction was carried out to determine which of the glutens, glutenins or gliadins, or both, were being digested. An SDS PAGE of the total gluten after increasing incubation time with the spPEP was performed (Figure 8). With 60 µg of spPEP and 400 µg of total gluten, both the glutenins and gliadins had been partially digested. The high molecular weight (HMW) glutenins were digested first, followed by the gliadins, showing that both are substrates for the spPEP. Within this timeframe, not all of the gliadins were susceptible to enzyme digestion. Further studies are underway to identify the specific glutens cleaved by the enzyme and for determination of the enzyme recognition sequence within the respective glutens.

## 4. Conclusions

The primary focus of the present study includes obtaining the complete sequence of the PEP cDNA, cloning and expression of a recombinant PEP. The initial experiments performed for the isolation of total RNA, enrichment of mRNA and cDNA synthesis suggested that mRNA coding for the sunn pest PEP is considerably low or nil in the nymphs and overwintering adults. Lack of PEP in the overwintering adults was expected, as the overwintered adults do not feed. The nymphs feed on grains, so it was expected to find some levels of PEP cDNA present; however, none of the PCR reactions using the nymph cDNA resulted in any amplicons using any of the primers. The spPEP enzyme appears to be expressed in high amounts only in the actively feeding adults.

The sequence results showed the spPEP to be at most 56% identical to other known PEP amino acid sequences. The spPEP amino acid sequence was most similar to the *Daphnia pulex*, as evidenced on the phylogenetic tree, and least similar to the bacterial PEPs, which were the most divergent. The mammalian PEPs were separated by several nodes on the tree from the spPEP, but were included in the top 25 hits obtained from BLASTX. The presence of a 5ꞌ UTR might be important in regulation of protein expression and is being further investigated. Upon translation of the ORF sequence, the highly conserved catalytic triad SDH is present, indicating that the sunn pest PEP might act similarly to other PEPs. Homology modeling showed that the spPEP could accommodate a tertiary structure almost identical to the porcine brain PEP known to hydrolyze oligopeptides shorter than 30 amino acids in length. The reason for the ability of the spPEP to cleave high molecular weight peptides, such as the glutenins and gliadins, still remains unexplained. The recombinant enzyme shows recognition of both the GPpNA peptide and its natural substrates, the glutenins and gliadins. Now that active recombinant spPEP can be expressed and purified, investigations to identify inhibitors specific for the spPEP can begin. Additionally, the enzyme can be purified in large enough amounts to determine the three-dimensional structure of the enzyme in the presence of inhibitors, peptides and its natural substrates, the glutens. Future studies are underway to investigate how this PEP can be so similar in catalytic activity of short peptides as other PEPs, yet be so unique in recognizing gluten proteins ranging in molecular weights up to 140 kD.

## Supplementary Files

Supplementary File 1
