# Supplementary material for: Cloning, Expression, Sequence Analysis and Homology Modeling of the Prolyl Endoprotease from Eurygaster integriceps Puton
Source: Insects. 2014 Oct 22;5(4):762–82. doi: 10.3390/insects5040762 (PMC4592603; doi:10.3390/insects5040762)
Supplement: Supplementary File 1 [file insects-05-00762-s001.pdf]

Supplementary Materials

Figure S1. Complete cDNA sequence of the Eurygaster integriceps prolyl endoprotease. The boxed nucleotides represent the 5' UTR. The coding region begins at nucleotide 330 and ends 2483 bp.

```
        10        20        30        40        50        60        70        80        90       100
AGTGGTATCAACGCAGAGTACATGGGGATTTGTCTAACATATTAACCGTTGTTCTAGTCTATTACAACGTTCCAGTTACCTCAAACAGTTACCTAGTTTG

       110       120       130       140       150       160       170       180       190       200
TCGGTCAATTTATCTAGCCGAGGAAGATTTCTCAAGGTTAGGACATGATCTAAAGAGAAACGGGATTTTTTTTTTCGAATTCAAATATACCTCAGTATG

       210       220       230       240       250       260       270       280       290       300
GTTCACATCGAGCTGAATCCTTTTGTGAAATGAAAGTTTGTTGAGAAAGCGATAATTAGGGTTTAAATAAAGCGAGTGATTGTATTGCTTTTATTTATTA

       310       320       330       340       350       360       370       380       390       400
TTTTTATTTTTTGCTTAGTCATACACCAAATGCCAAATACTGAAAAGATGAAAAAGTTCCAATACCCTGAAGCTCGGAGAGATGAAACTATCAAAGAAAC

       410       420       430       440       450       460       470       480       490       500
ATTTTTTGGAATCGAGGTTGCTGACCCATATAGATGGTTAGAAGATCCAGACAGCGAAGAGACCAAAAATTTTGTTGATGCCCAAAACAGTATTTCCGAA

       510       520       530       540       550       560       570       580       590       600
CCATATTTAAAGGGATGTCCGGCAAGGGATAAGATTAAAGCCAGGCTAACTCAGATGTTGGATTATCCTAAATATTCTCCTCCAGAAAAAGAAGGCAATC

       610       620       630       640       650       660       670       680       690       700
ATTACTTCTATTTTAAGAATACTGGTCTCCAGAATCATAGTGTTCTTTATATGCAAGACTCGTTAGATGGTCCGTCAAAAGTGTTCCTTGACCCTAACAC

       710       720       730       740       750       760       770       780       790       800
TTTTTCTTCGGATGGAACCGTTGCTTTGACTAGTACGTCGTTTTCAGAAGATGGATCGATAATGGGATACACCGTCAGTAAAAGTGGTTCTGATTGGTGT

       810       820       830       840       850       860       870       880       890       900
ACCATCCATTTCAGGAGAGTCGATACAGGTGAAGATTATCCGGAAGAATTGAAGTTTGTGAAATTTGGACATGCTGCTTGGACACACGATAATTTGGGAG

       910       920       930       940       950       960       970       980       990      1000
TTTTTTATACACGTTTTCCTGAAGTCGAAGGGAAGAGTGACGGTTCCGAAACCAGTCAAAACAGGAATCAAAAGATCTATTATCATAAAGTCGGTACACC

      1010      1020      1030      1040      1050      1060      1070      1080      1090      1100
ACAGTCTGAAGATATTCTTGTTGTCGAGCTGGATGATCCTGAATATATATACACAGTGTGCGTTAGCGATTGTGGAAGAGGGGTCGTAATTCTGCCTTCT

      1110      1120      1130      1140      1150      1160      1170      1180      1190      1200
AAATTTTGCCACAACAACTTGGTATATTTTTCGGATCTGTCAACCCTCAAAGACGGCATCAAGGGAAAGCTTGATGTTACTTGTATCGTGGATAAATTTG

      1210      1220      1230      1240      1250      1260      1270      1280      1290      1300
AAGCTGATTATGAGTTTGTTGCGAATACTGGAAGTAAATTTGTGTTCAGAACGAATAAAAATGCACACAACTACAAGCTGGTCGTCATCGATTTTGAAAA

      1310      1320      1330      1340      1350      1360      1370      1380      1390      1400
TCACAGCGAAGAAAATTGGGTGACGTTAGTTCCAGAGCACCCAACCGATGTGCTGGAACAGGCTGTTAGTGTCGCTCAAGATAAGCTGGTACTCTGTTAC

      1410      1420      1430      1440      1450      1460      1470      1480      1490      1500
ATTCGTGATGTCAAGAATACCCTCGATATTCACAGTTTGGTGGATGGATCTTTAATAAGGAAAATTCCGGTGCCTATAGGGACAGTAAGCTCGATATCTG

      1510      1520      1530      1540      1550      1560      1570      1580      1590      1600
GATCCAAGAAGCATAGCGAAGTCTTTTATACGTTCATTTCATTTACTAGCCCTGGCACTATCTACAGGTGTGATCTCAGTCAGTCGCCAATCCCTGATCC

      1610      1620      1630      1640      1650      1660      1670      1680      1690      1700
GGAGGTTTTTAGACAGATTACAATACCGGGCTATGATCCTTCAATGTTTGAAGAGAAACAAGTTTTCTATCTGAGTAAAGACGGCACTAGGATCCCCATG

      1710      1720      1730      1740      1750      1760      1770      1780      1790      1800
TTCCTCGTCCATAAAAAAGTCCTGGAACAAAACGGAAAAAAACCCTTGCCTTGTTTATGGGTACGGAGGCTTCAACATCAGCTTGCTTCCAATGTTCTCCA

      1810      1820      1830      1840      1850      1860      1870      1880      1890      1900
CTATCAGGCTCGTTTTTGTTCAGTATTTCAATGCTGTATTCGCTTCTGCAAACATAAGGGGAGGAGGGGAATATGGAGAAAAATGGCATGATGGTGGAAG
```

Figure S1. *Cont.*

```
         1910      1920      1930      1940      1950      1960      1970      1980      1990      2000
....|....|....|....|....|....|....|....|....|....|....|....|....|....|....|....|....|....|....|....|
ATTATTGAACAAGCAGAATTCCTTCGATGATTTTATTGCTGCTGGAGAATATTTAATCGCCGAGAAATACACCAATAAGAGTTGTCTGGCGATTCAGGGA

         2010      2020      2030      2040      2050      2060      2070      2080      2090      2100
....|....|....|....|....|....|....|....|....|....|....|....|....|....|....|....|....|....|....|....|
GCTTCTAATGGAGGCCTCCTCATTGGTGCATCGGTTAAACCCCCCTACTGGTTGCTGGAGGATCCTGAATATATATACACAGTGTGCGTTAGCGATTGTG

         2110      2120      2130      2140      2150      2160      2170      2180      2190      2200
....|....|....|....|....|....|....|....|....|....|....|....|....|....|....|....|....|....|....|....|
GAAGATGGGCAGTAATTCTGCCTTCTAAATTTTGCTACAACAACTTGGTATATTTTTCCGTTTTGTTCCAGGAGGAGCATTTCAAAAACGTCCTCAAGTA

         2210      2220      2230      2240      2250      2260      2270      2280      2290      2300
....|....|....|....|....|....|....|....|....|....|....|....|....|....|....|....|....|....|....|....|
TTCTCCTCTCCATAACATCAGAGTACCTGAAGATCAGTATCCAGCCTTGCTACTTCTAACTGCTTCTCACGACGATAGAGTCGTACCGCTGCACTCACTT

         2310      2320      2330      2340      2350      2360      2370      2380      2390      2400
....|....|....|....|....|....|....|....|....|....|....|....|....|....|....|....|....|....|....|....|
AAATATATCGCACAATTGCAACATGTAATGAGGGACAATCCTAAACAGGAAAATCCTCTCTTGATCCAAGTTGAAACGAAAGCTGGTCACGGCGCAGGCA

         2410      2420      2430      2440      2450      2460      2470      2480      2490      2500
....|....|....|....|....|....|....|....|....|....|....|....|....|....|....|....|....|....|....|....|
AACCCACAAATAAAAGAATCGAGGAACAAGTCGATATCCTTTGCTTCTTGATGAATTCAATGAATTTAAAATTTATAGAATAAGTTGTTTGTCAGATCAT

         2510      2520      2530      2540      2550      2560      2570      2580      2590      2600
....|....|....|....|....|....|....|....|....|....|....|....|....|....|....|....|....|....|....|....|
TTGACAACATTATCTGTGCTGCAAAATGTTTCTATACCTGAAAATAGTAACGATGGTCAACGCTGGGTGATTTCCAGTATATTTATTGTATTTTAGGATA

         2610      2620      2630      2640      2650      2660      2670      2680      2690      2700
....|....|....|....|....|....|....|....|....|....|....|....|....|....|....|....|....|....|....|....|
AAAGTTGAGTGACAGATGTAATGATACTTTCCCATATTGTTGCCATATTCCTTATCGGAACAGTTTTATTTGTATTGTACTATATTACTGTATCAAGTAT

         2710      2720      2730      2740      2750      2760      2770      2780      2790      2800
....|....|....|....|....|....|....|....|....|....|....|....|....|....|....|....|....|....|....|....|
TTACATCTTGCTTCCTATTTTTAAATTAAATATCATTTATTAACAAAAAAAAAAAAAAAAAAAAAAAAAAAAAAGTACTCTGCGTTGATACCACTGCTTG

         2810      2820
....|....|....|....|..
CCCTATAGTGAGTCGTATTAGA
```

Figure S2. Schematic of the 5' UTR transcription factor binding sites.

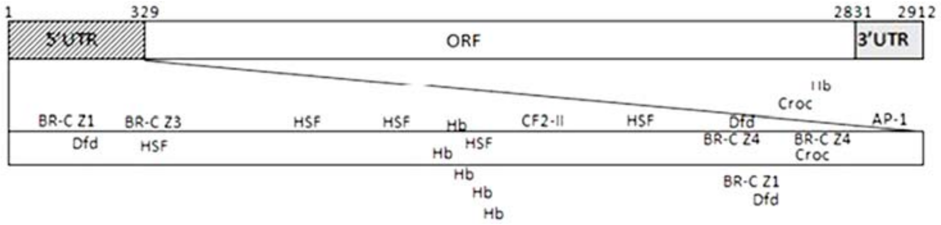

Figure S3. ClustalW2 analysis of top 25 hits resulting from BLASTX with the addition of the insects used to design degenerate primers if not present in the top 25 and three bacterial PEPs, *Elizabethkingia meningoseptica*, *Sphingomonas capsulata*, and *Myxococcus xanthus*.

```
NP_610129.3[Drosophila       ------------------------------------------------MS 2
XP_005177748.1[Musca         --------------------------------------------------
ETN62890.1[Anopheles         --------------------------------------------------
KFB39311.1[Anopheles         --------------------------------------------------
XP_001659779.1[Aedes         MISLLRRRQFLSASGSLRIVLQQQRDSFLSPLPQQLQSSKQRPLKTSAAA 50
XP_001843671.1[Culex         --------------------------------------------------
ACI03586.2[Eurygaster        --------------------------------------------------
EFX79244.1[Daphnia           --------------------------------------------------
NP_001004050.1[Sus           --------------------------------------------------
XP_006061999.1[Bubalus       --------------------------------------------------
NP_001244843.1[Macaca        --------------------------------------------------
NP_112614.1[Rattus           --------------------------------------------------
XP_005368679.1[Microtus      --------------------------------------------------
NP_035286.1[Mus              --------------------------------------------------
XP_005154718.1[Melopsittacus --------------------------------------------------
XP_005518296.1[Pseudopodoces --------------------------------------------------
```

Figure S3. *Cont.*

```
NP_001006410.1[Gallus        --------------------------------------------------
XP_009046664.1[Lottia        --------------------------------------------------
XP_005095067.1[Aplysia       --------------------------------------------------
EFN66352.1[Camponotus        --------------------------------------------------
EZA56288.1[Cerapachys        --------------------------------------------------
EFN76622.1[Harpegnathos      -----------MGKRYQKVYQHRRDNAKDCQHLHKNALTVFSINVGKGTR 39
XP_003394688.1[Bombus        -----------MAMFLIANISTKICSRKIQTGIYKNLLSKRPQFICASAF 39
XP_395364.2[Apis             --------------------------------------------------
XP_001603578.2[Nasonia       --------------------------------------------------
XP_008556849.1[Microplitis   --------------------------------------------------
KDR21572.1[Zootermopsis      --------------------------------------------------
ERL94303.1[Dendroctonus      --------------------------------------------------
XP_008193477.1[Tribolium     --------------------------------------------------
AAA24925.1[Elizabethkingia   -----------------------------------------------MKY 3
pdb|1YR2|[Sphingomonas       ------------------------------------MKNRLWLAMAAPLA 14
AAD31004.1[Myxococcus        --------------------------------------------------

NP_610129.3[Drosophila       RSDALVQSPTDLSRSVEEASLRIVYPEARKDGRFEEMIHGYKIKDVYRWL 52
XP_005177748.1[Musca         ----------------------LKYPVARKDESVVETHHGVEVKDVYRWL 28
ETN62890.1[Anopheles         ---------------TEG-HKVPQYPEARRDDSVADEFHGVKIADPYRWL 34
KFB39311.1[Anopheles         ---------------------RFNYPEARRDESVVDEFHGVKIADPYRWL 29
XP_001659779.1[Aedes         PSPTSKFSRIAMPEAQEC-AASFRYPEARRDDSIKEEIHGVTIPDPYRWL 99
XP_001843671.1[Culex         -----------MPEAQEAGTTAFQYPVARRDDSVVDEIHGQQIADPYRWL 39
ACI03586.2[Eurygaster        -------------------MKKFQYPEARRDETIKETFFGIEVADPYRWL 31
EFX79244.1[Daphnia           -------------------MSSFTYPIAKR-TDFSENLHGIAVEDPYRWL 30
NP_001004050.1[Sus           -------------------MLSFQYPDVYRDETAIQDYHGHKVCDPYAWL 31
XP_006061999.1[Bubalus       -------------------MLSFQYPDVYRDETAVQDYHGHKICDPYAWL 31
NP_001244843.1[Macaca        -------------------MLSLQYPDVYRDETAVQDYHGHKICDPYAWL 31
NP_112614.1[Rattus           -------------------MLSFQYPDVYRDETSVQDYHGHKICDPYAWL 31
XP_005368679.1[Microtus      -------------------MLSFQYPDVYRDETSVQDYHGHKICDPYAWL 31
NP_035286.1[Mus              -------------------MLSFQYPDVYRDETSVQEYHGHKICDPYSWL 31
XP_005154718.1[Melopsittacus --------------PPLPAMQAFQYPAVYRDETAVLDYHGCKISDPYCWL 36
XP_005518296.1[Pseudopodoces --------------PPQPVMQAFQYPEVYRDETAVSDYHGCKISDPYCWL 36
NP_001006410.1[Gallus        -------------------MQAFQYPEVYRDEAAVLDYHGHQISDPYCWL 31
XP_009046664.1[Lottia        -------------------MGVFKYPEVRRDETVVEEYHGTKVEDPYRWL 31
XP_005095067.1[Aplysia       -------------------MGKFQYPTPRRDETVADNYHGQEVKDPYRWM 31
EFN66352.1[Camponotus        -------------------MTRIQYPEARRDESVVDNYHGVEIADPYRWL 31
EZA56288.1[Cerapachys        -------------------MTRIQYPEARRDDSVVDNYHGVEITDPYRWL 31
EFN76622.1[Harpegnathos      KSLEATNYREQKDKGQSEVMTRIQYSMARRDESIVDDYHGTEIADPYRWL 89
XP_003394688.1[Bombus        STVKVLDPTIRKHIENHKIMEKLQYPEAYRDETIVDNYHGVEVQDSYRWL 89
XP_395364.2[Apis             -----------------KKMEKLQYPEAYRDESIIDNYHGIEVQDPYRWL 33
XP_001603578.2[Nasonia       ---------------------KFTYPKARRDETAVDVYHGVEIKDPYKWL 29
XP_008556849.1[Microplitis   --------------------KMFKYPEARRD-NTKDVYHGVEIQDPYRWL 29
KDR21572.1[Zootermopsis      -----------------QKQMKLIYPIARRDEDVVDNYHGHKILDPYRWL 33
ERL94303.1[Dendroctonus      ----------------------FTYPKPRRDETVKDDFFGTQVSDPYRWL 28
XP_008193477.1[Tribolium     ----------------TKRNMSFKYPDARRDETVKDNYFGTEITDPYRWL 34
AAA24925.1[Elizabethkingia   KKLSVAVAAFAFAAVSAQNSNSLKYPETKK-VNHTDTYFGNQVSDPYRWL 52
pdb|1YR2|[Sphingomonas       LATPVAFAQTPPTLAKDQAMSLPPYPASPQVPLVEDHFGEKVSDPWRWL 64
AAD31004.1[Myxococcus        -----------------------MSYPATRAEQVVDTLHGVQVADPYRWL 27
                                                                   .*  : * : *:

NP_610129.3[Drosophila       EDPD--SVDTQQFVNAQNNISQSFLERSAERE--NINSKLTKLWNFPKYG 98
XP_005177748.1[Musca         EDPD--AEETQKYVEEQNKISQPFLEGCESWK--KINEKLTKLWNYEKYG 74
ETN62890.1[Anopheles         EDPD--SEETREYVERQNEISKPFLDTCPEWK--KLNEKLRKRWNYPKYS 80
KFB39311.1[Anopheles         EDPD--SEETQAYVEKQNEISKPFLDTCPEWK--KLNEKLRKRWNYPKYS 75
XP_001659779.1[Aedes         EDPD--AEETQAYVEKQNEISKPFLDSCEQWK--ILNEKLTKRWNYPKYS 145
XP_001843671.1[Culex         EDPD--AEETQAYVEQQNKIAQPFLESCDEWK--KLNAKLTKRWNYPKYS 85
ACI03586.2[Eurygaster        EDPD--SEETKNFVDAQNSISEPYLKGCPARD--KIKARLTQMLDYPKYS 77
EFX79244.1[Daphnia           EDPD--SAETQEFVRLQNELTTPYIQGSPALS--SIKTRLTELWNFPKYS 76
NP_001004050.1[Sus           EDPD--SEQTKAFVEAQNKITVPFLEQCPIRG--LYKERMTELYDYPKYS 77
XP_006061999.1[Bubalus       EDPD--SEQTKAFVEAQNKITVPFLEQCPIRG--LYKERMTELYDYPKYS 77
NP_001244843.1[Macaca        EDPD--SEQTKAFVEAQNKITVPFLEQCPIRG--LYKERMTELYDYPKYS 77
NP_112614.1[Rattus           EDPD--SEQTKAFVEAQNKITVPFLEQCPIRG--LYKERMTELYDYPKYS 77
XP_005368679.1[Microtus      EDPD--SEQTKAFVEAQNKITVPFLEQCPIRG--LYKERMTELYDYPKYS 77
NP_035286.1[Mus              EDPD--SEQTKAFVEAQNKITVPFLEQCPIRG--LYKERMTELYDYPKYS 77
XP_005154718.1[Melopsittacus EDPD--SEQTKAFVEAQNKLTVPFLEQCPVRG--LFKERMTELYDYPKYS 82
```

Figure S3. *Cont.*

```
XP_005518296.1[Pseudopodoces EDPD--SEQTKAFVEAQNKLTVPFLEQCPVRG--LFKERMTELYDYPKYS 82
NP_001006410.1[Gallus        EDPD--SEQTKAFVEAQNKLTVPFLEQCPVRG--LFKERMTELYDYPKYS 77
XP_009046664.1[Lottia        EDPD--SEETKAFVEAQNKISEPFLEKCPVKE--KIKNRITDIWDYPKYG 77
XP_005095067.1[Aplysia       EDPD--GEETKAFVDAQNSISRPFINACPVKE--QIQKRITEVWDYPKYG 77
EFN66352.1[Camponotus        EDPD--STETKAFVDAQNAITKPYLAACKVRD--SIHERLTQLWDFPKYS 77
EZA56288.1[Cerapachys        EDPD--SAETKAFVDAQNAITKPYLAACKARD--NIHERLTQLWDFPKYS 77
EFN76622.1[Harpegnathos      EDPD--SEETKAFVEAQNAITKPYLASCSVRN--NIHKRLKQLWDFPKYS 135
XP_003394688.1[Bombus        EDPD--SEKTKAFVDAQNSVTIPYLASCKARQ--DIHDRLKQLWDFPKYS 135
XP_395364.2[Apis             EDPD--SEKTKAFVDAQNSITTPYLTSCKARQ--DIHDRLKQLWDFPKYS 79
XP_001603578.2[Nasonia       EDPE--SEETKAYVDAQNAITVPFIQACPKRQ--AIHDRLKQLWDYPKYS 75
XP_008556849.1[Microplitis   EDPE--ADEVKAFVDKQNALSRPFLSTCDSVDPEVILERLKQLWDFPKYS 77
KDR21572.1[Zootermopsis      EDPD--SPNTSKFVDEQNALTRPILENCSSRS--DILARLTELWNFPKYS 79
ERL94303.1[Dendroctonus      EDPD--SEETRSFVNAQNEISRPYLENCPYKD--SIRSRITQLWDFMRFS 74
XP_008193477.1[Tribolium     EDPD--SEETKKYVDGQNAVTRPYLDGCSFKE--SIKKKITQLWNYPKFS 80
AAA24925.1[Elizabethkingia   EDDR--AEDTKAWVQQEVKFTQDYLAQIPFRG--QIKKQLLDIWNYEKIS 98
pdb|1YR2|[Sphingomonas       EADVRTDAKVAAWVQAQSAYTAAYLKQLPERA--ALEKRMKALIDYERFG 112
AAD31004.1[Myxococcus        EDEK--APEVQTWMTAQNAHAREALAKFPGRE--ALAARFKELFYTDSVS 73
                             *       ..  ::  :   :   :             ::         .

NP_610129.3[Drosophila       CPMRHGNYYYFFKNTGLQNQSVLMQQKT---LESPESIFLDTNSISSDGT 145
XP_005177748.1[Musca         CPMKHGKYYYFYKNSGLQNQSVMYQQDS---LDGEPRLFFDPNALSSDGT 121
ETN62890.1[Anopheles         CPFKHGNKYFFFMNTGLQNQDVLYVQDS---LKGEPKVFLDPNTLSTDGT 127
KFB39311.1[Anopheles         CPFKHGNKYFFFMNTGLQNQDVLYVQDK---LDGEPRVFLDPNTLSTDGT 122
XP_001659779.1[Aedes         CPFKHGSRYFFFMNTGLQNQDVLYVQNS---LEDEPKVFLDPNALSKDGT 192
XP_001843671.1[Culex         CPFKHASRYFFFMNTGLQNQDVLYVQNS---LDDEPKVFLDPNALSKDGT 132
ACI03586.2[Eurygaster        PPEKEGNHYFYFKNTGLQNHSVLYMQDS---LDGPSKVFLDPNTFSSDGT 124
EFX79244.1[Daphnia           CPTKKGNHYFFYKNSGLQNHSVLFVQDS---LESEPRIFLDPNTLSDDGT 123
NP_001004050.1[Sus           CHFKKGKRYFYFYNTGLQNQRVLYVQDS---LEGEARVFLDPNILSDDGT 124
XP_006061999.1[Bubalus       CHFKKGKRYFYFYNTGLQNQRVLYVQDS---LEGEARVFLDPNTLSDDGT 124
NP_001244843.1[Macaca        CHFKKGKRYFYFYNTGLQNQRVLYVQDS---LEGDARVFLDPNILSDDGT 124
NP_112614.1[Rattus           CHFKKGKRYFYFYNTGLQNQRVLYVQDS---LEGEARVFLDPNTLSDDGT 124
XP_005368679.1[Microtus      CHFKKGKRYFYFYNTGLQNQRVLYVQDS---LEGEARVFLDPNTLSDDGT 124
NP_035286.1[Mus              CHFKKGKRYFYFYNTGLQNQRVLYVQDS---LEGEARVFLDPNTLSDDGT 124
XP_005154718.1[Melopsittacus CHFKKGKRYFHFYNTGLQNQRVLYVQDS---LDADAKVFLDPNKLSDDGT 129
XP_005518296.1[Pseudopodoces CHFKKGKRYFHFYNTGLQNQRVLYVQDS---LDAEAKVFLDPNKLSDDGT 129
NP_001006410.1[Gallus        CHFKKGKRYFHFYNTGLQNQRVLYVQDS---LDADAKVFLDPNKLSDDGT 124
XP_009046664.1[Lottia        CPRKHGDHFYYSYNTGLQNQSVTYVQDS---LEAEARVFLDPNKLSEDGT 124
XP_005095067.1[Aplysia       CPKKHGDHYYYFHNSGLQNQSVMYVQDS---LDAEPRVFLDPNKLSDDGT 124
EFN66352.1[Camponotus        CPAKHGGKYYFYKNTGLQNQSVLYVQDT---LESEPKVFLDPNTLSEDGT 124
EZA56288.1[Cerapachys        CPAKHGGKYYFYKNTGLQNQSVLYVQDT---LESEPKVFFDPNTLSEDGT 124
EFN76622.1[Harpegnathos      CPAKYGGKYYFYKNTGLQNQSVLYVQDT---LESEPRVFLDPNTFSEDGT 182
XP_003394688.1[Bombus        CPARYGNKYYFYKNTGLQNQSVLYVQDT---LDSEPRIFLDPNTFSEDGT 182
XP_395364.2[Apis             CPARYGNKYYFYKNTGLQNQSVLYVQDT---LDSEPRVFLDPNTLSEDGT 126
XP_001603578.2[Nasonia       CPAKKGSKYYFFMNTGLQNQSVFYVQDS---LDGEPRVFLDPNTFSTDGT 122
XP_008556849.1[Microplitis   CPRKHGDKYFFYKNTGLQNQSVIYSQNSPTDSEEEAKVFFDPNTLSDDGS 127
KDR21572.1[Zootermopsis      CPYRHGDKYFFYMNTGLQNQSVLYIQDS---LDSKPRVFLDPNLLSDDGT 126
ERL94303.1[Dendroctonus      VPFKHGNKYFQYRNTGLQNQSVLYVQDS---LKGKERVFLDLNGFSEDGT 121
XP_008193477.1[Tribolium     TPYRHGTKYYQYRNTGLQNQSVIYVQKD---LASKAEIFLDPNTFSEDGT 127
AAA24925.1[Elizabethkingia   APFKKGKYTYFYKNDGLQAQSVLYRKDA----SGKTEVFLDPNKFSDKGT 144
pdb|1YR2|[Sphingomonas       LPQRRGASVFYSWNSGLMNQSQLLVRPADAPVGTKGRVLLDPNTWAKDGA 162
AAD31004.1[Myxococcus        TPSRRNGRFFYVRTHKDKEKAILYWRQGES---GQEKVLLDPNGWSKDGT 120
                               :     :   .     :     :           :::* *  : .*:

NP_610129.3[Drosophila       TAISHIKFSEDGAFMAYGLSESGSDWNKIRIRNTKEG-IDLPEILEKVKF 194
XP_005177748.1[Musca         IALAQKSFSEDGKYMAYGLSASGSDWIKIYIRDVETG-KDQEEVLEKVKF 170
ETN62890.1[Anopheles         IALVGSRFSDDGQLYAYGLSQSGSDWTKLKIRNVETG-EDFPETIEHTKF 176
KFB39311.1[Anopheles         IALVGSRFSDDGQLYAYGLSQSGSDWTKLKIRNVETG-EDFPETIEHTKF 171
XP_001659779.1[Aedes         IALVGSRFSDDGSLFAYGLSQSGSDWTKLKVRNVSTG-EDFPETLEHTKF 241
XP_001843671.1[Culex         IALVGSRFSDDGNLFAYGLSQSGSDWTKLKVRDVNTG-EDFPETIEHTKF 181
ACI03586.2[Eurygaster        VALTSTSFSEDGSIMGYTVSKSGSDWCTIHFRRVDTG-EDYPEELKFVKF 173
EFX79244.1[Daphnia           VSLSMKKFSEDGEIFAYGLSQSGSDWNSIHFKCVKTG-EDFPEVLEKIKF 172
NP_001004050.1[Sus           VALRGYAFSEDGEYFAYGLSASGSDWVTIKFMKVDGA-KELPDVLERVKF 173
XP_006061999.1[Bubalus       VALRGYAFSEDGEYFAYGLSASGSDWVTIKFMKVDGA-KELPDVLERVKF 173
NP_001244843.1[Macaca        VALRGYAFSEDGEYFAYGLSASGSDWVTIKFMKVDGA-KELPDVLERVKF 173
NP_112614.1[Rattus           VALRGYAFSEDGEYFAYGLSASGSDWVTIKFMKVDGA-KELPDVLERVKF 173
XP_005368679.1[Microtus      VALRGYAFSEDGEYFAYGLSASGSDWVTIKFMKVDGA-KELPDVLERVKF 173
NP_035286.1[Mus              VALRGYAFSEDGEYFAYGLSASGSDWVTIKFMKVDGA-KELPDVLERVKF 173
```

Figure S3. *Cont.*

```
XP_005154718.1[Melopsittacus VALRGYAFSEDGEYFAYGLSSSGSDWVTIKFMKVEGP-EDLPDTLERVKF 178
XP_005518296.1[Pseudopodoces VALRGYAFSEDGEYFAYGLSSSGSDWITIKFMKVEGP-EDLPDTLERVKF 178
NP_001006410.1[Gallus        VALRGYAFSEDGEYFAYGLSSSGSDWVTIKFMKVEGA-EELPDTLERVKF 173
XP_009046664.1[Lottia        ISLRGSSFTEDGSVYAYGLSKSGSDWISIHFKKAPSG-EDLPDVLERCKF 173
XP_005095067.1[Aplysia       VSLRGSAFTENGLTLAYGLSKSGSDWITVKFKKAPSG-EDLPDVLEQVKF 173
EFN66352.1[Camponotus        VAITSSSFSEDGSIFAYGLSESGSDWSTIHFLNAQTG-EKYPEILEKVKF 173
EZA56288.1[Cerapachys        VAISSSSFSEDGNIFAYGLSKSGSDWSTIHFLNTQTG-EKYPEVLEKVKF 173
EFN76622.1[Harpegnathos      VAITSSSFSEDGSIFAYGLSKSGSDWSTIHFLNAETG-EKYPEILEKVKF 231
XP_003394688.1[Bombus        IAITSSKFSEDGSIYAYGLSISGSDWCTIHFMNTETG-EKYPEILEKVKF 231
XP_395364.2[Apis             IAITISKFSEDGSIYAFGLSASGSDWCTIHFMNTETG-EKYPEILEKVKF 175
XP_001603578.2[Nasonia       VAISSGEFSEDGGIYAYALSASGSDWNTIHFINTKTG-EKYPEVLEKVKY 171
XP_008556849.1[Microplitis   VAISNTEFSKDGSIFAYGLSKSGSDWSEIHFKNVNTG-ENYPEVLEKIKY 176
KDR21572.1[Zootermopsis      VALSRTRFSEDGKILAYGLSSSGSDWVTIHFKLVDTG-EDYPEVLEKVKF 175
ERL94303.1[Dendroctonus      VALSGNCFSDDGSTFAYGLSSSGSDWIEIRFRDVETG-EDHPETLKKVKY 170
XP_008193477.1[Tribolium     VALSGTAFSEDGQTFAYGLSSSGSDWLEIKFKDVETG-KDYKEILKKVKF 176
AAA24925.1[Elizabethkingia   TSLANLSFNKKGTLVAYSISEGGSDWNKIIILDAETK-KQIDETLLDVKF 193
pdb|1YR2|[Sphingomonas       TALDAWAASDDGRLLAYSVQDGGSDWRTVKFVGVADG-KPLADELKWVKF 211
AAD31004.1[Myxococcus        VSLGTWAVSWDGKKVAFAQKPNAADEAVLHVIDVDSGEWSKVDVIEGGKY 170
                              ::     . .*   .:  . ..:*   : .  .        : :   *:

NP_610129.3[Drosophila       SNVSWTKDSKGFFYGRYTDQDGIFDGSETK-LAENQKLYYHLLGESPHQD 243
XP_005177748.1[Musca         SEISWTKDNKGFFYGRYPNQEGKTDGSETK-SNENQKLYYHYVGQPQEKD 219
ETN62890.1[Anopheles         VTASWTKDNKGFFYARYPVVDGKADGSETA-ANENQKLYYHRVGDSQDKD 225
KFB39311.1[Anopheles         VTASWTKDNKGFFYARYPAVDGKADGSETA-ANENQKLYYHRVGESQDKD 220
XP_001659779.1[Aedes         VTASWAKDNKGFFYARYPVVDGKADGSETA-ANENQKLYFHRVGDSQDKD 290
XP_001843671.1[Culex         VTASWTKDNKGFFYARYPVVEGKADGSETA-ANENQKLYYHRIGEPQDKD 230
ACI03586.2[Eurygaster        GHAAWTHDNLGVFYTRFPEVEGKSDGSETS-QNRNQKIYYHKVGTPQSED 222
EFX79244.1[Daphnia           SSISWTHDRKGVFYSCYPEQQGKTDGSETT-SNENHKLFYHRIGTQQSED 221
NP_001004050.1[Sus           SCMAWTHDGKGMFYNAYPQQDGKSDGTETS-TNLHQKLYYHVLGTDQSED 222
XP_006061999.1[Bubalus       SCMAWTHDGKGMFYNAYPQQDGKSDGTETS-TNLHQKLCYHVLGTDQSED 222
NP_001244843.1[Macaca        SCMAWTHDGKGMFYNSYPQQDGKSDGTETS-TNLHQKLYYHVLGTDQSED 222
NP_112614.1[Rattus           TCMAWTHDGKGMFYNSYPQQDGKSDGTETS-TNLHQKLCYHVLGTDQSED 222
XP_005368679.1[Microtus      TCMAWTHDGKGMFYNSYPQQDGKSDGTETS-TNLHQKLCYHVLGTDQSED 222
NP_035286.1[Mus              TCMAWTHDGKGMFYNSYPQQDGKSDGTETS-TNLHQKLCYHVLGTDQSED 222
XP_005154718.1[Melopsittacus SCMAWTHDGKGMFYNCYPTQDGKSDGTETS-TNLHQKLHYHVLGTNQSED 227
XP_005518296.1[Pseudopodoces SCMAWTHDGKGMFYNCYPEQDGKSDGTETS-TNLHQKLHYHVLGTNQSED 227
NP_001006410.1[Gallus        SCMAWTHDGKGMFYNCYPKQDGKSDGTETS-TNLHQKLHYHVLGTNQSED 222
XP_009046664.1[Lottia        SSMAWMHDHTGFFYNSYPEQDGKTDGTETT-TNLHQKLYYHKLGTKQSDD 222
XP_005095067.1[Aplysia       SSMAWTHDHTGFFYNSYPNQEGKVDGTETT-SNLHQKLYFHRLGTEQTAD 222
EFN66352.1[Camponotus        SPITWTHDNRGIFYGQYRDQQGKTDGSETL-GNQNQKLCYHIVGTSQSDD 222
EZA56288.1[Cerapachys        SPITWTHDNRGIFYGQYRDQQGKTDGSETL-GNQNQKLCYHIVGTSQADD 222
EFN76622.1[Harpegnathos      SPITWTHDNRGIFYGQYRDQQGKTDGSETL-GNQNQKLCYHVVGTSQSDD 280
XP_003394688.1[Bombus        SPITWTHDNRGIFYGCYPDQKGKTDGSETE-GNRDQKLCYHIVGTPQSED 280
XP_395364.2[Apis             SPITWTHDNYGIFYGCYPDQKGKTDGSETE-GNRDQKLCYHIIGTPQSED 224
XP_001603578.2[Nasonia       SSITWTHDNVGVFYACYPEQLEKADGSETF-VNKNQKLCYHKVGTPQSED 220
XP_008556849.1[Microplitis   STIAWTHDNKGIFYGTYLEQQGIVDGSETL-KARDQKLCYHKIATKQSED 225
KDR21572.1[Zootermopsis      SSMTWTHDNEGLFYGRYPDQVGKADGSETV-GMQHQKLYYHRVGTPQSED 224
ERL94303.1[Dendroctonus      SPMTWMHDNKGFFYGGYLNQTGKADGSETT-SSENQKLYYHELGTDQSQD 219
XP_008193477.1[Tribolium     SPMTWMHDNKGFFYGAYLDQTGKADGSETK-TNENQKLYYHELGTDQSQD 225
AAA24925.1[Elizabethkingia   SGISWLGD-EGFFYSSYDKPK---DGSVLSGMTDKHKVYFHKLGTKQSQD 239
pdb|1YR2|[Sphingomonas       SGLAWLGNDALLYSRFAEPKEGQAFQALNY----NQTVWLHRLGTPQSAD 257
AAD31004.1[Myxococcus        ATPKWTPDSKGFYYEWLPTDPSIKVDERPG----YTTIRYHTLGTEPSKD 216
                                 *  :   .:                       .:  * :.     *

NP_610129.3[Drosophila       ILIAEFPEHPSWRFKTDISDCGKYLILSISHTVR-DNMLYYAELGS--EE 290
XP_005177748.1[Musca         VLIAEFPEEPTWRIQSVVSDCGKYLILAIVKDCR-DNIVYYADLEP--GA 266
ETN62890.1[Anopheles         VLIAEFPEEPSWRLMPEVSDCGKYLMLFIMKGCK-DMLLYFSNLEQ--AG 272
KFB39311.1[Anopheles         VLIAEFPEEPSWRLMPEVSDCGKYLMLFIMKGCK-DMLLYFSNLEK--AG 267
XP_001659779.1[Aedes         VLIAEFPEEPSWRLMPEVSDCGKYLMLFIMKGCK-DMLLYFSKLES--SD 337
XP_001843671.1[Culex         VLIAEFPEEPSWRLMPEVSDCGKYLMLFIMKGCK-DMLLYFSNLEK--AG 277
ACI03586.2[Eurygaster        ILVVELDD-PEYIYTVCVSDCGRGVVILPSKFCH-NNLVYFSDLSTLK-D 269
EFX79244.1[Daphnia           ILVVEFSEEPKWRIQGGVTDCGRYLIVTTGRDCQ-YNNVYFCDLTALPNQ 270
NP_001004050.1[Sus           ILCAEFPDEPKWMGGAELSDDGRYVLLSIREGCDPVNRLWYCDLQQ-ESN 271
XP_006061999.1[Bubalus       ILCAEFPDEPKWMGGAELSDDGRYVLLSIREGCDPVNRLWYCDLHQ-EPN 271
NP_001244843.1[Macaca        ILCAEFPDEPKWMGGAELSDDGRYVLLSIREGCDPVNRLWYCDLQQ-ESN 271
NP_112614.1[Rattus           VLCAEFPDEPKWMGGAELSDDGRYVLLSIWEGCDPVNRLWYCDLQQ-GSN 271
XP_005368679.1[Microtus      VLCAEFPDEPKWMGGAELSDDGRYVLLSIWEGCDPVNRLWYCDLQQ-ESN 271
```

Figure S3. *Cont.*

```
NP_035286.1[Mus              ILCAEFPDEPKWMGGAELSDDGRYVLLSIWEGCDPVNRLWYCDLQQ-EPN 271
XP_005154718.1[Melopsittacus ILCAEFPDEPKWMGGAEVSDDGRYVLLSIREGCDPVNRLWYCDLQK-ESQ 276
XP_005518296.1[Pseudopodoces ILCAEFPDEPKWMGGAEVSDDGRYVLLSVREGCDPVNRLWYCDLQK-ESQ 276
NP_001006410.1[Gallus        ILCAEFPDEPKWMGGAEISDDGRYVLLSIREGCDPVNRLWYCDLQK-ESQ 271
XP_009046664.1[Lottia        ILVAEFPDHPKWMTGCEISDCGRYILIYVREGCEPVNRLFYVDIQT-LKN 271
XP_005095067.1[Aplysia       VLVGEMPDFPKWMIGAEVSDCGRYLLLTPSEGCDPVNRLFYVDLES-LEG 271
EFN66352.1[Camponotus        VVAIEFPEEPLWRIGAEVSDCGNWLIVTPLKDCR-DNLVYFTPLK--AGM 269
EZA56288.1[Cerapachys        VIAVEFPEEPLYRIGAEVSDCGNWLIVTPLKDCR-DNLVYFTPLK--AGM 269
EFN76622.1[Harpegnathos      VVAVEFPEEPLWRIGAQVSDCGNWLIITPLKDCR-DNLVFFTPLK--AEM 327
XP_003394688.1[Bombus        VIVVEFPEEPLWRIGAQVSDCGKWLIVTPVKDCR-DNLVYFTELK--PEK 327
XP_395364.2[Apis             IIVVEFPEEPLWRIGAQVSDCGKWLIVTPVKDCR-DNLVYFTELK--PEM 271
XP_001603578.2[Nasonia       VVVVDFPEHPLWRIDAKVTDCGRWLVVMPQQECR-DNLVFFAKLN--TAE 267
XP_008556849.1[Microplitis   VIAVEFPEEPLWRISSQVSDCGEYLIVSPRKDCR-DNLVYFTKLP--KDN 272
KDR21572.1[Zootermopsis      ILVVEFPEEPLWRIDAEVSDCGQWLIVMPQKDCR-DNLLFFCDLKSLPDN 273
ERL94303.1[Dendroctonus      VVVVEFED-KELRMGACVSHCGTYLVITPIKGCK-NNLLYFARIDP--TN 265
XP_008193477.1[Tribolium     VVVVEFDD-PHLRIGAHVSHCGKYLVITGTKGCK-NNLLYFAQLD---SG 270
AAA24925.1[Elizabethkingia   ELIIGGDKFPRRYLSGYVTEDQRYLVVSAANATN-GNELYIKDLKN---- 284
pdb|1YR2|[Sphingomonas       QPVFATPELPKRGHGASVSSDGRWVVITSSEGTDPVNTVHVARVTNG--- 304
AAD31004.1[Myxococcus        TVVHERTGDPTTFLQSDLSRDGKYLFVYILRGWS-ENDVYWKRPGE---- 261
                                              ::     :.:   .       :

NP_610129.3[Drosophila       KNA--FQLELKPIVDKFEADFD----------------------YITNEG 316
XP_005177748.1[Musca         EIT--GKLQVKKIVTKFESDYD----------------------YITNTG 292
ETN62890.1[Anopheles         GLS--GKLDFVKVVTEFDSDYD----------------------YVTNEG 298
KFB39311.1[Anopheles         NLE--SKLDFVKVVTEFDSDYD----------------------YVTNEG 293
XP_001659779.1[Aedes         NIT--GKLDFVKVVTEFDSDYD----------------------YITNEG 363
XP_001843671.1[Culex         GIT--GKLDFTKIVTEFDSDYDVSQCPQTSQINQQSHNSTAPFQYITNEG 325
ACI03586.2[Eurygaster        GIK--GKLDVTCIVDKFEADYE----------------------FVANTG 295
EFX79244.1[Daphnia           AIS--GKLELTTVVDKMEADYE----------------------YVTNTG 296
NP_001004050.1[Sus           GIT--GILKWVKLIDNFEGEYD----------------------YVTNEG 297
XP_006061999.1[Bubalus       GIT--GILKWVKLIDNFEGEYD----------------------YVTNEG 297
NP_001244843.1[Macaca        GIT--GILKWVKLIDNFEGEYD----------------------YVTNEG 297
NP_112614.1[Rattus           GIN--GILKWVKLIDNFEGEYD----------------------YITNEG 297
XP_005368679.1[Microtus      GIT--GILKWVKLIDNFEGEYD----------------------YVTNEG 297
NP_035286.1[Mus              GIT--GILKWVKLIDNFEGEYD----------------------YVTNEG 297
XP_005154718.1[Melopsittacus GIS--GILQWVKLIDNFEAEYE----------------------YVTNEG 302
XP_005518296.1[Pseudopodoces GIT--GILQWVKLIDNFEAEYE----------------------YVTNEG 302
NP_001006410.1[Gallus        GIS--GILQWVKLIDNFEAEYE----------------------YVTNEG 297
XP_009046664.1[Lottia        GID--GKLPYVKVVDNFDAEYE----------------------YITNEG 297
XP_005095067.1[Aplysia       GIS--GILPYVKIVDNFDAEYQ----------------------YITNEG 297
EFN66352.1[Camponotus        DIS--NNLPLTQVVDKLEADYE----------------------YITNVG 295
EZA56288.1[Cerapachys        TIS--NNLPLTQVVDKLEADYE----------------------YVTNIG 295
EFN76622.1[Harpegnathos      KIT--TNLPLTQVVDVLEADYE----------------------YVTNVG 353
XP_003394688.1[Bombus        KIA--EKLQLTQVVDKLEADYE----------------------YVTNDD 353
XP_395364.2[Apis             KIR--EKLHLTQVVDKLEADYE----------------------YVTNDD 297
XP_001603578.2[Nasonia       GIK--GKLPLTEVVGNLEADYE----------------------YVTNVG 293
XP_008556849.1[Microplitis   KIDNYKNLNLIKVVDKFEADYD----------------------YVTNDG 300
KDR21572.1[Zootermopsis      KIS--GKLNLTQVVYKLEADYE----------------------YVTNEG 299
ERL94303.1[Dendroctonus      RIN--KPLELTPVVTTFDADYK----------------------YITNVG 291
XP_008193477.1[Tribolium     KIT--GKLKLTEVVTEFVADFE----------------------YITNDK 296
AAA24925.1[Elizabethkingia   ------KTDFIPIITGFESNVG----------------------LVDTDG 306
pdb|1YR2|[Sphingomonas       -----KIGPVTALIPDLKAQWDF----------------------VDGVG 327
AAD31004.1[Myxococcus        -------KDFRLLVKGVGAKYE----------------------VHAWK 281
                                         ::  . ..                         :

NP_610129.3[Drosophila       SNLYFHTNKDAPNYRVIVIDVNNPAEEHWTTPIPEHKKDVLEWAKCVDGN 366
XP_005177748.1[Musca         SKVYFRTNKDASNYRVIMIDFENPAQENWQTLIPEHATDVLDWVHCVNED 342
ETN62890.1[Anopheles         SIFSFRTNKGAPNYRIVNIDFNEPSLENWKTLVPEHPKNVLDWTTCVNKD 348
KFB39311.1[Anopheles         SIFSFRTNKGAPNYRIVNIDFDQPAMEHWKTLVAEHPKNVLDWTTCVNKD 343
XP_001659779.1[Aedes         SIFSFRTNKGAPNYRVVNIDFDSPAMDNWKTLIEEHPKNVLDWSTCVNKD 413
XP_001843671.1[Culex         SIFSFRTNKGAPNYRVVNIDFDEPALDKWTTLIAEDPKNVLDWSSCVNGD 375
ACI03586.2[Eurygaster        SKFVFRTNKNAHNYKLVVIDFENHSEENWVTLVPEHPTDVLEQAVSVAQD 345
EFX79244.1[Daphnia           AVVVFRTNKDAPNYRLIQIDFNQPEREQWKTLLEADPSDVLDWVACINKD 346
NP_001004050.1[Sus           TVFTFKTNRHSPNYRLINIDFTDPEESKWKVLVPEHEKDVLEWVACVRSN 347
XP_006061999.1[Bubalus       TVFTFKTNRHSPNYRLINIDFTDPEESRWKVLVPEHEKDVLEWVACVRSN 347
NP_001244843.1[Macaca        TVFTFKTNRHSPNYRVINIDFRDPEESKWKVLVPEHEKDVLEWVACVRSN 347
NP_112614.1[Rattus           TVFTFKTNRNSPNYRLINIDFTDPDESKWKVLVPEHEKDVLEWVACVRSN 347
```

Figure S3. *Cont.*

```
XP_005368679.1[Microtus      TVFTFKTNRNSPNYRLINIDFMDPDESKWKVLVPEHEKDVLEWVACVRSN 347
NP_035286.1[Mus              TVFTFKTNRNSPNYRLINIDFTDPDESKWKVLVPEHEKDVLEWVACVRSN 347
XP_005154718.1[Melopsittacus TVFTFKTNRHSPNYRLINIDFSDPGESKWKVLVPEHERDVLEWAACVRSN 352
XP_005518296.1[Pseudopodoces TVFTFKTNRHSPNYRLINIDFSDPEESKWKVLVPEHEKDVLEWVACVRSN 352
NP_001006410.1[Gallus        TVFTFKTNRHSPNYRLINIDFSDPEESKWKVLIPEHERDVLEWVACVRSN 347
XP_009046664.1[Lottia        TVFTFKTNLKAPKYKLINIDFNQPEMAKWSTLVEEDEKSVLEWVGCVNQN 347
XP_005095067.1[Aplysia       PVFTFKTNLKAPNYKLINIDLTKPEPENWKTLVEEDASAVLEWATCVNND 347
EFN66352.1[Camponotus        TKAVFRTNKNAPNYKLIAIDLLDYGQDKWVDLLPEHPENVLDWADAVDGD 345
EZA56288.1[Cerapachys        TKAVFRTNKNAPNYKLIAIDLLDYGEDKWVDLLPEHSENVLDWATAVDGD 345
EFN76622.1[Harpegnathos      TKAVFRTNKNAPNYKLIAIDLLDHGQDKWVDLLPEHPENVLDWATAVDGD 403
XP_003394688.1[Bombus        TKAIFRTNKNAPNYKLIAIDLLDYKQEKWVDLLPEHPDNVLDWACAVDGD 403
XP_395364.2[Apis             TKAIFRTNKNAPNYKLIAIDLLDYKQEKWVDLLPEHPDNVLDWACAVDGD 347
XP_001603578.2[Nasonia       TKAVFRTNKNAPNFKLIATDFENYQENSWSELIAEHSRNVLDWATAVDKD 343
XP_008556849.1[Microplitis   AQMIFSTNRNAPNYRLVRINFEDYAEEKWTDLIPEDPKRVLDWALAVHGD 350
KDR21572.1[Zootermopsis      PICIFRTNKNAPNYHLIKIDFTNPSQENWTTLVPEHEKDVLDWASAVKND 349
ERL94303.1[Dendroctonus      SKFYFRTNKDAMNYRIIIINFNNPSESEWTNLIAEHPKDVLHDVKVINKT 341
XP_008193477.1[Tribolium     NLFYFHTNKNASNYRIVIIDFDNPKESEWKDLISEHPKDVLDWAHAINEN 346
AAA24925.1[Elizabethkingia   DTLFLHTDKNAPNMRMVKTTIQNPKPETWKDVIAETSEPMR---VNSGGG 353
pdb|1YR2|[Sphingomonas       DQLWFVSGDGAPLKKIVRVDLSG-STPRFDTVVPE-SKDNLESVGIAGN- 374
AAD31004.1[Myxococcus        DRFYVLTDEGAPRQRVFEVDPAKPARASWKEIVPEDSSASLLSVSIVGG- 330
                                 . :.  :   ::.           :   :

NP_610129.3[Drosophila       KLVVCYNCDVKHILQARDLSTGKLIRQFGLD-IGSINGISGKKSNSEIFY 415
XP_005177748.1[Musca         KLVLGYIQDVKSALQVNSLQTGELIHKFDLD-IGTIVALSGKKKESELFY 391
ETN62890.1[Anopheles         RIVLGYIDDVKSLLQVHSLADGSFVSKFPLE-IGTVVGFSGKKKYSEIFY 397
KFB39311.1[Anopheles         RIVLGYIDDVKSLLQVHSLADGAFVSKFPLD-IGTVVGFSGKKKYSEIFY 392
XP_001659779.1[Aedes         RVVLGYINDVKSVLQVHSLHDGSFVSKFPLE-IGTVVGFSGKKKYSEIFY 462
XP_001843671.1[Culex         KIVLGYIDDVKSVLQVHSLQDGRFLSKFPLA-IGNVVGFSGKKKYSEIFY 424
ACI03586.2[Eurygaster        KLVLCYIRDVKNTLDIHSLVDGSLIRKIPVP-IGTVSSISGSKKHSEVFY 394
EFX79244.1[Daphnia           KLIVCYMHDVKNILQLRDLQNGQLLKTYALE-MGTVREFSGKNTSSEFFF 395
NP_001004050.1[Sus           FLVLCYLHDVKNTLQLHDLATGALLKIFPLE-VGSVVGYSGQKKDTEIFY 396
XP_006061999.1[Bubalus       FLVLCYLHDVKNTLQLHDLATGALLKTFPLE-VGSIVGYSGQKKDTEIFY 396
NP_001244843.1[Macaca        FLVLCYLHDVKNILQLHDLTTGALLKTFPLE-VGSIVGYSGQKKDTEIFY 396
NP_112614.1[Rattus           FLVLCYLRNVKNILQLHDLTTGALLKTFPLD-VGSVVGYSGRKKDSEIFY 396
XP_005368679.1[Microtus      FLVLCYLHDVKNILQLHDLTTGALLKTFPLD-VGSVVGYSGRKKDSEIFY 396
NP_035286.1[Mus              FLVLCYLHDVKNILQLHDLTTGALLKTFPLD-VGSVVGYSGRKKDSEIFY 396
XP_005154718.1[Melopsittacus FLVLCYLHDVKNILQLHDLATGAHLKTFPLE-VGSIVGYSGQKKDTEIFY 401
XP_005518296.1[Pseudopodoces FLVLCYLHDVKNILQLHDLATGAHLKTFPLE-VGSIVGYSGQKKDTEIFY 401
NP_001006410.1[Gallus        FLVLCYLHDVKNILQLHDLATGAHLKTFPLD-VGSIVGYSGQKKDNEIFY 396
XP_009046664.1[Lottia        KLVLCYLEDVKNKLYIHDLALGTRKAELPLD-VGTIVGYSGRKKNTEIFY 396
XP_005095067.1[Aplysia       RLVLCYLRDVKNELYVYDLASGKQNYQFPLN-VGSVAGFSGKKKGTEIFY 396
EFN66352.1[Camponotus        KFVACYIQDVKNILQLHCLKTGKIIRTFPLD-LGTVVGFSGEKKYSEIFY 394
EZA56288.1[Cerapachys        KFVACYIQDVKNILQLHCLKTGEVLRTFPLD-LGTVVGFSGEKKYSEIFY 394
EFN76622.1[Harpegnathos      KFVACYIQDVKNILQLHCLSTGNVLRTFPLD-LGTVVGFSGEKKYSEIFY 452
XP_003394688.1[Bombus        KFVACYIADVKNILQLHSLTSGEKLRIFPLD-VGTIVNFAGQKKYSEIFY 452
XP_395364.2[Apis             KFVACYIEHVKNILQLHSLKSGDILRTFPLD-VGTIVNFAGQKKYSEIFY 396
XP_001603578.2[Nasonia       KLVVCYIEDVKNVLGVHSLETGKLIRQLPLD-VGTVVGFSGDLKYSEIFY 392
XP_008556849.1[Microplitis   KLVVCYIQDVKHILELHCLKTGKLLKTFPLD-LGTIVGISGEREYSEIFY 399
KDR21572.1[Zootermopsis      KLVVCYIHDVKSVLQLHELNTGALLKTFPLS-VGTVTGYSGKKKHTEIFY 398
ERL94303.1[Dendroctonus      MLVICYLKDVKHTMHIFDINTGNKIYDFKLD-VGTVSEISGKRHHSEMFY 390
XP_008193477.1[Tribolium     MLVVCYLQDVKNIMQLYDIKSSNKLHDFKLD-VGTISAISGKKYHKEMFF 395
AAA24925.1[Elizabethkingia   YFFATYMKDALSQIKQYDK-TGKLVREIKLPGSGTAGGFGGEKTEKELYY 402
pdb|1YR2|[Sphingomonas       RLFASYIHDAKSQVLAFDL-DGKPAGAVSLPGIGSASGLSGRPGDRHAYL 423
AAD31004.1[Myxococcus        HLSLEYLKDATSEVRVATL-KGKPVRTVQLPGVGAASNLMGLEDLDDAYY 379
                              .   *  ..   :       .       :   *      *     . :

NP_610129.3[Drosophila       GFSSFLSPGIIFYYDFAKPS------------EKPTVLREISLNLEGFSR 453
XP_005177748.1[Musca         NFSSFLTPGTIYHYNFKWNN------------FTPKVLREIKLNLEGFSP 429
ETN62890.1[Anopheles         HFVSFLTPGIIYHYDFAADS---------TATVEPTIFREVKI--EDFDN 436
KFB39311.1[Anopheles         HFVSFLTPGIIYHYDFEGSAKEESKEGSTEAVMKPTIFREVKI--EDFDN 440
XP_001659779.1[Aedes         HFVSFLTPGIIYHYDFAKEG------------TEPKIFRQVKI--EDFDN 498
XP_001843671.1[Culex         HFVSFLTPGVIYHYDFAAGEG-----------AEAKIFRQVKI--EDFDD 461
ACI03586.2[Eurygaster        TFISFTSPGTIYRCDLSQSPI-----------PDPEVFRQITI--PGYDP 431
EFX79244.1[Daphnia           QFGSFLTPGVIYRCDIGESVE-----------AEPTVFRQIEL--NGFDP 432
NP_001004050.1[Sus           QFTSFLSPGIIYHCDLTK-EE-----------LEPRVFREVTV--KGIDA 432
XP_006061999.1[Bubalus       QFTSFLSPGIIYHCDLTK-EE-----------LEPRVFREVTV--KGIDA 432
NP_001244843.1[Macaca        QFTSFLSPGIIYHCDLTK-EE-----------LEPRVFREVTV--KGIDA 432
```

Figure S3. *Cont.*

```
NP_112614.1[Rattus           QFTSFLSPGVIYHCDLTR-EE-----------LEPRVFREVTV--KGIDA 432
XP_005368679.1[Microtus      QFTSFLSPGVIYHCDLTK-EE-----------LEPMVFREVTV--KGIDA 432
NP_035286.1[Mus              QFTSFLSPGVIYHCDLTK-EE-----------LEPMVFREVTV--KGIDA 432
XP_005154718.1[Melopsittacus QFTSFLSPGIIYHCDLTK-EE-----------LEPRVFREVTV--KGFDP 437
XP_005518296.1[Pseudopodoces QFTSFLSPGIIYHCDLTK-EE-----------LEPTVFREVTV--KGFDP 437
NP_001006410.1[Gallus        QFTSFLSPGIIYHCDLTK-EE-----------LEPRVFREVTV--KGFDP 432
XP_009046664.1[Lottia        QFMSFLTPGIIYRCDMTT-DN-----------YSPKVFREINV--KDFDV 432
XP_005095067.1[Aplysia       QFLSFLTPGVIYHCDMSG-RD-----------YQPKVFREITV--QGFDA 432
EFN66352.1[Camponotus        QFTSFLTPGIIYTIDLK--KE-----------EEPRILREIKV--KGFDA 429
EZA56288.1[Cerapachys        QFTSFLTPGIIYTIDLK--KE-----------QEPRVLREIKV--KGFDA 429
EFN76622.1[Harpegnathos      QFTSFLTPGIIYTIDLK--KE-----------EEPRVLREIKV--EHFDA 487
XP_003394688.1[Bombus        QFKSFLVPGIIYRVDLK--NE-----------EEPQVLREIKV--KNFDP 487
XP_395364.2[Apis             QFKSFLIPGIIYRVDLK--NE-----------EEPQVLREIKV--KNFDP 431
XP_001603578.2[Nasonia       QFTSFLTPGIIYTLDLKE-NE-----------EKPKVFREIKV--NDFDA 428
XP_008556849.1[Microplitis   QFTSILTPGKIFMVDLA--KE-----------EEPKMLREIKV--NGFDS 434
KDR21572.1[Zootermopsis      QFTSFLSPGIIYRCDLTK-SV-----------LEPEVFREIRV--HDFDA 434
ERL94303.1[Dendroctonus      SVCSFLTPSIIYRVQFNG-DQ-----------ITEERYYETKV--ADFDP 426
XP_008193477.1[Tribolium     SFCSFLTPNIIYKVDFDQ-GS-----------IKETLFHETKV--GDFES 431
AAA24925.1[Elizabethkingia   SFTNYITPPTIFKFSIDS--------------GKSEVYQKPKV---KFNP 435
pdb|1YR2|[Sphingomonas       SFSSFTQPATVLALDPAT--------------AKTTPWEPVHLT---FDP 456
AAD31004.1[Myxococcus        VFTSFTTPRQIYKTSVST--------------GKSELWAKVDVP---MNP 412
                              . .   *  :   .                           :     .

NP_610129.3[Drosophila       DNYSVEQVFYKSTDDTDIPMFIVQR--KRDIAEPRPCLLYGYGGFNYSLM 501
XP_005177748.1[Musca         SNYKVEQVFYESKDSTKIPMFIVYKNTKAEKRTPRPCFLYGYGGFNISMQ 479
ETN62890.1[Anopheles         SQYTVEQIFYHSKDGEKVPMFIVQR--KQKEKEHKPCLLYGYGGFNICVQ 484
KFB39311.1[Anopheles         SQYTVEQVFYHSKDGEKIPMFIVQR--RQDTKEHKPCLLYGYGGFNICVQ 488
XP_001659779.1[Aedes         SLYKVDQVFYESKDGERIPMFVVQR--KSDKQEKKPCLLYGYGGFNICIQ 546
XP_001843671.1[Culex         SLYKVEQVFYKSKDGERVPMFIVQK--KSDKQEKKPCLLYGYGGFNICIQ 509
ACI03586.2[Eurygaster        SMFEEKQVFYLSKDGTRIPMFLVHK-KVLEQNGKNPCLVYGYGGFNISLL 480
EFX79244.1[Daphnia           SLFETQQVFYPSKDGTRIPMFIVKK-KTVVLDGTNPCLMYGYGGFNISLE 481
NP_001004050.1[Sus           SDYQTVQIFYPSKDGTKIPMFIVHK-KGIKLDGSHPAFLYGYGGFNISIT 481
XP_006061999.1[Bubalus       SDYQTVQIFYPSKDGTKIPMFIVHK-KGIKLDGSHPAFLYGYGGFNISIT 481
NP_001244843.1[Macaca        SDYQTVQIFYLSKDGTKIPMFIVHK-KGIKLDGSHPAFLYGYGGFNISIT 481
NP_112614.1[Rattus           SDYQTIQVFYPSKDGTKIPMFIVHK-KGIKLDGSHPAFLYGYGGFNISIT 481
XP_005368679.1[Microtus      SDYQTIQVFYPSKDGTKIPMFIVHK-KGIKLDGSHPAFLYGYGGFNISIT 481
NP_035286.1[Mus              ADYQTIQIFYPSKDGTKIPMFIVHK-KGIKLDGSHPAFLYGYGGFNISIT 481
XP_005154718.1[Melopsittacus SVYQTIQVFYPSKDGTKIPMFIIHK-KGIKLDGSHPAFLYGYGGFNISIT 486
XP_005518296.1[Pseudopodoces SVYQTIQVFYPSKDGTKIPMFIIHK-KGIKLDGSHPAFLYGYGGFNISIT 486
NP_001006410.1[Gallus        SVYQTIQVFYPSKDGTKIPMFIIHK-KGIKLDGSHPAFLYGYGGFNISIT 481
XP_009046664.1[Lottia        SGFETKQVFYPSKDGTKIPMFIVHK-KGLNLDGSHPVLLYGYGGFSVSIS 481
XP_005095067.1[Aplysia       SLFETEQVFFKSTDGTKIPMFIVHK-KGLKKDGNNPTLLYGYGGFSISIT 481
EFN66352.1[Camponotus        SLYKTSQIFYPSKDGTKIPMFIVTK-SDAVLDGTMPALLYGYGGFNASIQ 478
EZA56288.1[Cerapachys        SLYKTSQIFYTSKDGTKIPMFIVTK-NDAVLDGKMPALLYGYGGFNASIQ 478
EFN76622.1[Harpegnathos      SLYKTSQIFYPSKDGTKIPMFIVTK-SDAVLDGTMPALLYGYGGFNASIQ 536
XP_003394688.1[Bombus        SLYKTSQIFYTSKDGTKIPMFIVMK-HDAVLDGSMPALLYGYGGFNVSIQ 536
XP_395364.2[Apis             SLYKTSQIFYTSKDGTKIPMFIVMK-HDAVLDGSMPALLYGYGGFNVSIQ 480
XP_001603578.2[Nasonia       SSYKTTQIFYSSKDGTKIPMFIVHK-KDLVLDGSSPALLYGYGGFNVSIQ 477
XP_008556849.1[Microplitis   TAYKMSQIFYSSKDGTKIPMFIVTR-TDAVLDGSLPALLYGYGGFNVSIY 483
KDR21572.1[Zootermopsis      SQYETKQVFYESKDGTKIPMFLVYK-TGLVRDGKQPCLLHGYGGFNVSIL 483
ERL94303.1[Dendroctonus      SLYETKQVFYKSKDGTNIPMFIINK-IGFVNDGSQPCLLYGYGGFNVNLT 475
XP_008193477.1[Tribolium     SKYETKQVFYKSKDGTEIPMFIINK-KGLVNDGSKPCLLYGYGGFNVNLT 480
AAA24925.1[Elizabethkingia   ENYVSEQVFYTSADGTKIPMMISNK-KGLKKDGKNPTILYSYGGFNISLQ 484
pdb|1YR2|[Sphingomonas       ADFRVEQVFYPSKDGTKVPMFIVRR-KDAK--GPLPTLLYGYGGFNVALT 503
AAD31004.1[Myxococcus        EQYQVEQVFYASKDGTKVPMFVVHR-KDLKRDGNAPTLLYGYGGFNVNME 461
                               :   *:*: * *.  :**::  :          * :::.****.  :

NP_610129.3[Drosophila       PSFGILSLMFMDTFDGVLAFPNLRGGGEYGMEWHNGGRMLSKQNVFNDFQ 551
XP_005177748.1[Musca         PSFGITGLMFIDTFDGVIAYPNLRGGGEYGEKWHNAGRLLNKQNVFNDFQ 529
ETN62890.1[Anopheles         PSFSITGLVFIDSFDGILAYPNIRGGGEYGERWHNSGRLLKKQNVFDDFQ 534
KFB39311.1[Anopheles         PSFSITGLVFIDSFDGILAYPNIRGGGEYGERWHNAGRLLKKQNVFDDFQ 538
XP_001659779.1[Aedes         PSFSITGLVFIDSFDGILAYPNIRGGGEYGERWHNAGRLLKKQNVFDDFQ 596
XP_001843671.1[Culex         PSFSITGLVFIDSFDGILAYPNIRGGGEYGERWHNAGRLLKKQNVFDDFQ 559
ACI03586.2[Eurygaster        PMFSTIRLVFVQYFNAVFASANIRGGGEYGEKWHDGGRLLNKQNSFDDFI 530
EFX79244.1[Daphnia           PAFSVTRIVFMQHFNGVFAVPNIRGGGEYGEAWHDGGRLFNKQNSFDDFH 531
NP_001004050.1[Sus           PNYSVSRLIFVRHMGGVLAVANIRGGGEYGETWHKGGILANKQNCFDDFQ 531
XP_006061999.1[Bubalus       PNYSVSRLIFVRHMGGVLAVANIRGGGEYGETWHKGGILANKQNCFDDFQ 531
```

Figure S3. *Cont.*

```
NP_001244843.1[Macaca        PNYSVSRLIFVRHMGGILAVANIRGGGEYGETWHKGGILANKQNCFDDFQ 531
NP_112614.1[Rattus           PNYSVSRLIFVRHMGGVLAVANIRGGGEYGETWHKGGILANKQNCFDDFQ 531
XP_005368679.1[Microtus      PNYSVSRLIFVRHMGGVLAVANIRGGGEYGETWHKGGILANKQNCFDDFQ 531
NP_035286.1[Mus              PNYSVSRLIFVRHMGGVLAVANIRGGGEYGETWHKGGILANKQNCFDDFQ 531
XP_005154718.1[Melopsittacus PSYSVSRLIFVRHLGGVLAVANIRGGGEYGETWHKGGILANKQNCFDDFQ 536
XP_005518296.1[Pseudopodoces PSYSVSRLIFVRHLGGVLAVANIRGGGEYGETWHKGGILANKQNCFDDFQ 536
NP_001006410.1[Gallus        PSYSVSRLIFVRHLGGVLAVANIRGGGEYGETWHKGGILANKQNCFDDFQ 531
XP_009046664.1[Lottia        PTFSPSRLVLLQHLNVVYALANIRGGGEYGETWHKAGCLGNKQNGFDDFQ 531
XP_005095067.1[Aplysia       PSFSPSRVVFLQHLGGVYALANIRGGGEYGETWHKDGSLGNKQNCFNDFQ 531
EFN66352.1[Camponotus        PTFSVTRLVFIQHLNGILAVANVRGGGEYGERWHNAGRFFNRQNVFDDFQ 528
EZA56288.1[Cerapachys        PTFSVTRLVFIQHLNGVLAVANVRGGGEYGEKWHNAGRFFNRQNVYDDFQ 528
EFN76622.1[Harpegnathos      PTFSVTRLVFIQHLDGVLAVANVRGGGEYGEKWHNAGRFFNRQNVFDDFQ 586
XP_003394688.1[Bombus        PTFSVTKLVFVQHLNGVLAVANIRGGGEYGEKWHNGGRFFNKQNVFDDFQ 586
XP_395364.2[Apis             PTFSVTKLVFVQHLNGVLAVANIRGGGEYGEKWHNGGRFFNKQNVFDDFQ 530
XP_001603578.2[Nasonia       PTFSVTRLVFLQHLNGVLAIPNIRGGGEYGEKWHNGGRFTNKQNVFDDFQ 527
XP_008556849.1[Microplitis   PTFSVMRLAFVQHLRGVFAIANIRGGGEYGEKWHNSGRLDNKQNVFDDFQ 533
KDR21572.1[Zootermopsis      PTFSVTHLVFIQHFGGILAVPNIRGGGEYGERWHNAGRLLNKQNGFDDFQ 533
ERL94303.1[Dendroctonus      PSFAISRLVFVGNFNGVYALANIRGGGEYGDNWHNGGRFANKQNCFDDFQ 525
XP_008193477.1[Tribolium     PSFGVSRLVFIENFDGVYALANIRGGGEYGDNWHNGGRFGKKQNCFDDFQ 530
AAA24925.1[Elizabethkingia   PAFSVVNAIWMEN-GGIYAVPNIRGGGEYGKKWHDAGTKQQKKNVFNDFI 533
pdb|1YR2|[Sphingomonas       PWFSAGFMTWIDS-GGAFALANLRGGGEYGDAWHDAGRRDKKQNVFDDFI 552
AAD31004.1[Myxococcus        ANFRSSILPWLDA-GGVYAVANLRGGGEYGKAWHDAGRLDKKQNVFDDFH 510
                             . :       :       * .*:*******  **. *   .::* ::**
NP_610129.3[Drosophila       AAAEFLTKNNYTTKDRLAIQGASNGGLLVGACIN------QRPD-LFGAA 594
XP_005177748.1[Musca         AAAEYLVANNYTTKDRLVIQGGSNGGLLVGACIN------QRPD-LFGAA 572
ETN62890.1[Anopheles         YAAQYLVEHGYTRHEKIAIQGGSNGGLLVGACIN------QRPD-LFGAA 577
KFB39311.1[Anopheles         YAAQFLVEQGYTRHEKIAIQGGSNGGLLVGACIN------QRPD-LFGAA 581
XP_001659779.1[Aedes         HAAQFLVESGYTTHDQIVIQGGSNGGLLVGACIN------QRPD-LFGAA 639
XP_001843671.1[Culex         YAAQYLVESGYTSHDQIVIQGGSNGGLLVGACIN------QRPD-LFGAA 602
ACI03586.2[Eurygaster        AAGEYLIAEKYTNKSCLAIQGASNGGLLIGASVKPPYWLLEDPEYIYTVC 580
EFX79244.1[Daphnia           SAAEYLIANGYTSSSKLAIQGASNGGLLIGACVN------QRPE-LYAAG 574
NP_001004050.1[Sus           CAAEYLIKEGYTSPKRLTINGGSNGGLLVATCAN------QRPD-LFGCV 574
XP_006061999.1[Bubalus       CAAEYLIKEGYTSPKMLTINGGSNGGLLVATCAN------QRPD-LFGCV 574
NP_001244843.1[Macaca        CAAEYLIKEGYTSPKRLTINGGSNGGLLVAACAN------QRPD-LFGCV 574
NP_112614.1[Rattus           CAAEYLIKEGYTTSKRLTINGGSNGGLLVAACAN------QRPD-LFGCV 574
XP_005368679.1[Microtus      CAAEYLVKEGYTSPKRLTINGGSNGGLLVAVCAN------QRPD-LFGCV 574
NP_035286.1[Mus              CAAEYLIKEGYTSPKRLTINGGSNGGLLVAACAN------QRPD-LFGCV 574
XP_005154718.1[Melopsittacus CAAKYLIKEGYTSPKKLTINGGSNGGLLVAACAN------QRPD-LFGCA 579
XP_005518296.1[Pseudopodoces CAAKYLIKEGYTSPKKLTINGGSNGGLLVAACAN------QRPD-LFGCA 579
NP_001006410.1[Gallus        YAAKYLIREGYTAPKKLTINGGSNGGLLVAACAN------QRPD-LFGCV 574
XP_009046664.1[Lottia        SAAQYLIDNKYTSPNRIAIQGGSNGGLLVAACSN------QRPD-LFGAA 574
XP_005095067.1[Aplysia       SAAEYLVAQGYTSSKKLVINGGSNGGLLVGACLN------QRPD-LFAGG 574
EFN66352.1[Camponotus        YAAKYLVENGYTTSTKLTIQGGSNGGLAVAVCIN------QRPD-LFGAA 571
EZA56288.1[Cerapachys        YAAKYLVENGYTTSAKLTIQGGSNGGLAVAVCIN------QRPD-LFGAA 571
EFN76622.1[Harpegnathos      YAAKYLIENGYTSSAKLTIQGGSNGGLAVATCIN------QRPD-LFGAA 629
XP_003394688.1[Bombus        AAAEYLVEKGYTTSSKLSILGASNGGLLVAACVN------QRPD-LFGAA 629
XP_395364.2[Apis             TAAEYLIENGYTTSSKLSILGASNGGLLIAACIN------QRPD-LFGAA 573
XP_001603578.2[Nasonia       CAAEYLIDNRYTSPKKLIIQGGSNGGLLVGACIN------QRPD-LFGAA 570
XP_008556849.1[Microplitis   SAAEYLIANKYTEAKKLTIHGGSNGGLLVAACIN------QRPE-LYGAA 576
KDR21572.1[Zootermopsis      SAAEYLLKEGYTCREKLTIRGGSNGGLLVAACIN------QRPD-LFGAA 576
ERL94303.1[Dendroctonus      HAAKYLIEENYTSASKLTIQGGSNGGLLVAACIN------QAPE-LFGAA 568
XP_008193477.1[Tribolium     YAAKYLVENKYTKVDKLIIQGGSNGGLLVAACIN------QAPE-LFGAA 573
AAA24925.1[Elizabethkingia   AAGEYLQKNGYTSKDYMALSGRSNGGLLVGATMT------MRPD-LAKVA 576
pdb|1YR2|[Sphingomonas       AAGEWLIANGVTPRHGLAIEGGSNGGLLIGAVTN------QRPD-LFAAA 595
AAD31004.1[Myxococcus        AAAEYLVQQKYTQPKRLAIYGGSNGGLLVGAAMT------QRPE-LYGAV 553
                              *.::*     *    : : * ***** :..  .        *: :

NP_610129.3[Drosophila       VAQVGVMDMLRFHKFTIGHAWCSDYGNPDE-KVHFANLIKFSPLHNVHIP 643
XP_005177748.1[Musca         VAQVGVMDMLRFHKFTIGHAWCSDYGNPDE-KEHFENLIKYSPLHNVHTP 621
ETN62890.1[Anopheles         IAQVGVMDMLRFHKFTIGRAWVSDYGDLNE-KDHFENLLRYSPLHNVRRP 626
KFB39311.1[Anopheles         IAQVGVMDMLRFHKFTIGRAWVSDYGDLNE-KDHFENLLRYSPLHNVRTP 630
XP_001659779.1[Aedes         IAQVGVMDMLRFHKFTIGRAWVSDYGDIDE-KEHFENLYKYSPLHNVHTP 688
XP_001843671.1[Culex         VAHVGVMDMLRFHKFTIGRAWVSDYGDITE-KEHFENLLRYSPLHNVHTP 651
ACI03586.2[Eurygaster        VSDCGRWAVILPSKFCYNN--LVYFSVLFQ-EEHFKNVLKYSPLHNIRVP 627
EFX79244.1[Daphnia           IAHVGVMDMLRFHKFTVGYCWVSDYGSPEE-KAAFENLLKFSPLHNLKVP 623
NP_001004050.1[Sus           IAQVGVMDMLKFHKYTIGHAWTTDYGCSDS-KQHFEWLIKYSPLHNVKLP 623
XP_006061999.1[Bubalus       IAQVGVMDMLKFHKYTIGHAWTTDYGCSDN-KQHFEWLIKYSPLHNVKLP 623
```

Figure S3. *Cont.*

```
NP_001244843.1[Macaca        IAQVGVMDMLKFHKYTIGHAWTTDYGCSDS-KQHFEWLVKYSPLHNVKLP 623
NP_112614.1[Rattus           IAQVGVMDMLKFHKFTIGHAWTTDYGCSDS-KQHFEWLLKYSPLHNVKLP 623
XP_005368679.1[Microtus      IAQVGVMDMLKFHKFTIGHAWTTDYGCSDS-KQHFEWLLKYSPLHNVKLP 623
NP_035286.1[Mus              IAQVGVMDMLKFHKFTIGHAWTTDYGCSDT-KQHFEWLLKYSPLHNVKLP 623
XP_005154718.1[Melopsittacus IAQVGVMDMLKFHKYTIGHAWTTDYGCSDC-KEQFEWLFKYSPLHNIKLP 628
XP_005518296.1[Pseudopodoces IAQVGVMDMLKFHKYTIGHAWTTDYGCSDC-KEQFEWLHKYSPLHNVKLP 628
NP_001006410.1[Gallus        IAQVGAMDMLKFHKYTIGHAWTTDYGCSDH-KEQFEWLCKYSPLHNVKLP 623
XP_009046664.1[Lottia        IGQVGVLDMLRFHKFTIGHAWTTDYGCSDK-PEDFQWLYKYSPLHNINVP 623
XP_005095067.1[Aplysia       IAQVGVLDMLRFHKFTIGHAWITDYGSSDD-PEQFKWLIKYSPLH--NIP 621
EFN66352.1[Camponotus        IAQVGVMDMLRFHKFTIGSAWVSDYGSSDD-AKHFQNLLKYSPLHNVRIP 620
EZA56288.1[Cerapachys        IAQVGVMDMLRFHKFTIGSAWVSDYGSSDD-LKHFQNLLKYSPLHNVRIP 620
EFN76622.1[Harpegnathos      IAQVGVMDMLRFHKFTIGSAWVSDYGSADD-PKHFQNLLKYSPLHNVKVP 678
XP_003394688.1[Bombus        IAQVGVMDMLRFHKFTIGVAWVSDYGSSDD-PKHFENLLKYSPLHNVRIP 678
XP_395364.2[Apis             IAQVGVMDMLRFHKFTIGVAWVSDYGSSDD-SKHFENLIKYSPLHNVRVP 622
XP_001603578.2[Nasonia       IAQVGVMDMLKFHKFTVGYAWTSDYGSSDD-REHFKNLLKYSPLHNVKPP 619
XP_008556849.1[Microplitis   IADVGVMDMLRFHKFTIGYAWISDYGSSDD-DKQFKTLLKYSPLHNVKVP 625
KDR21572.1[Zootermopsis      IVQVGVLDMLRFHKFTIGYSWVSDYGSSDE-KEHYENLIKYSPLHNVKVP 625
ERL94303.1[Dendroctonus      ICQVGVLDMLRYHKFTIGYAWKSDYGCSDE-EDEFKYLYKYSPLHNITVP 617
XP_008193477.1[Tribolium     ICQVGVLDMLRFHKFTIGYAWKSDYGCSEE-QEQFEYLYKYSPLHNIRVP 622
AAA24925.1[Elizabethkingia   FPGVGVLDMLRYNKFTAGAGWAYDYGTAEDSKEMFEYLKSYSPVHNVKAG 626
pdb|1YR2|[Sphingomonas       SPAVGVMDMLRFDQFTAGRYWVDDYGYPEK-EADWRVLRRYSPYHNVRSG 644
AAD31004.1[Myxococcus        VCAVPLLDMVRYHLFGSGRTWIPEYGTAEK-PEDFKTLHAYSPYHHVRPD 602
                                     ::    :  .      :.        :  :  :** *

NP_610129.3[Drosophila       LNPNQEYPSTLILTADHDDRVSPLHSYKFVAALQEAVRYSEYQLNPILLR 693
XP_005177748.1[Musca         KSPSEEYPSTLVLTADHDDRVSPLHSLKFIAALQEAVRNSEYQKNPILLR 671
ETN62890.1[Anopheles         TSEKEQYPATLVLTADHDDRVSPLHSLKFVAALHHAINGCEHQRNPLLLR 676
KFB39311.1[Anopheles         AAEKEQYPATLVLTADHDDRVSPLHSLKFMAALHQAVKDCEHQKNPLLLR 680
XP_001659779.1[Aedes         KSEKEQYPATLVLTADHDDRVSPLHSLKFVAALHHAIKDSEHQKNPLLLR 738
XP_001843671.1[Culex         SSEREQYPATLVLTADHDDRVSPLHSLKFMAALHHAVRDSKYQKNPLLLR 701
ACI03586.2[Eurygaster        ED---QYPALLLLTASHDDRVVPLHSLKYIAQLQHVMRDNPKQENPLLIQ 674
EFX79244.1[Daphnia           ETG--QYPAMLLLTADHDDRVVPLHSLKYMAQMHHTFRDCPKQTNPLMIR 671
NP_001004050.1[Sus           EADDIQYPSMLLLTADHDDRVVPLHSLKFIATLQYIVGRSRKQNNPLLIH 673
XP_006061999.1[Bubalus       EADDIQYPSMLLLTADHDDRVVPLHSLKFIATLQHLVGRSRKQNNPLLIH 673
NP_001244843.1[Macaca        EADDIQYPSMLLLTADHDDRVVPLHSLKFIATLQYIVGRSRKQSNPLLIH 673
NP_112614.1[Rattus           EADDIQYPSMLLLTADHDDRVVPLHSLKFIATLQYIVGRSRKQSNPLLIH 673
XP_005368679.1[Microtus      EADDIQYPSMLLLTADHDDRVVPLHSLKFIATLQYIVGRSRKQSNPLLIH 673
NP_035286.1[Mus              EADDIQYPSMLLLTADHDDRVVPLHSLKFIATLQYIVGRSRKQSNPLLIH 673
XP_005154718.1[Melopsittacus EEDGIQYPSTLLLTADHDDRVVPLHSLKFIATLQYVVGRSSKQTNPLLIH 678
XP_005518296.1[Pseudopodoces EEDGIQYPSTLLLTADHDDRVVPLHSLKFIATLQYIVGRSSKQTNPLLIH 678
NP_001006410.1[Gallus        EEDGIQYPATLLLTADHDDRVVPLHSLKFIATLQYVVGRSRKQTNPLLIH 673
XP_009046664.1[Lottia        DGN-IQYPSMLLLTGDHDDRVVPLHSLKYIAEVQRKVGSTEKQKNPLIIR 672
XP_005095067.1[Aplysia       KGV-EQYPALLLLTGDHDDRVVPLHSLKFIAELQSVVGSSDTQTNPVIIR 670
EFN66352.1[Camponotus        P-DDVQYPATLLLTADHDDRVVPLHTLKLIATLQYTFRNVSKQENPILAR 669
EZA56288.1[Cerapachys        A-DDTQYPAMLLLTADHDDRVVPLHTLKLTATLQHTLGNVPKQENPLLAR 669
EFN76622.1[Harpegnathos      Q-DDIQYPATLLLTADHDDRVVPLHTLKLIATLQYTLGNVPKQTNPLLAR 727
XP_003394688.1[Bombus        --ENGQYPATLLLTADHDDRVVPLHSLKLIATLQCTLGKLPQQTNPLLIK 726
XP_395364.2[Apis             --ENGQYPATLLLTADHDDRVVPLHSLKLIATLQYTLGKLPQQTNPLLIK 670
XP_001603578.2[Nasonia       K-DGGQYPATLLLTADHDDRVVPLHSLKLIATLQHEIGSLPQQTNPILIR 668
XP_008556849.1[Microplitis   A-NDVQYPATLLLTADHDDRVVPLHTLKLIATLQHVLGKLPQQTNPILAR 674
KDR21572.1[Zootermopsis      HGDIIQYPATLLLTADHDDRVVPLHSLKFIATLHHVLRNCSDQTNPLLIR 675
ERL94303.1[Dendroctonus      K-NGAQYPATLLLTADHDDRVVPLHSLKFCAELQEKIGNLPQQENPLLIR 666
XP_008193477.1[Tribolium     Q-NGGQYPATLLLTADHDDRVVPLHSLKFIAELQNKIGRLPTQKNPLMIR 671
AAA24925.1[Elizabethkingia   T----CYPSTMVITSDHDDRVVPAHSFKFGAELQAKQAC----KNPVLIR 668
pdb|1YR2|[Sphingomonas       ----VDYPAILVTTADTDDRVVPGHSFKYTAALQ----TAAIGPKPHLIR 686
AAD31004.1[Myxococcus        ----VRYPALLMMAADHDDRVDPMHARKFVAAVQ----NSPGNPATALLR 644
                                   **: :: :.. **** * *: *  * ::           . : :

NP_610129.3[Drosophila       VYTKAGHGAGKPTKMRISEATDIITFFKKTLNVDCINL------------ 731
XP_005177748.1[Musca         VYSKAGHGAGKPTSKRIEEATDVLTFMLRSLNI----------------- 704
ETN62890.1[Anopheles         VYSKAGHGMGKPTAKKIEESTDILTFMYKTLGLKL--------------- 711
KFB39311.1[Anopheles         VYSKAGHGMGKPTAKKIEEATDILTFMYKTLGLKL--------------- 715
XP_001659779.1[Aedes         VYSKAGHGMGKPTAKKIEEATDILTFMYKTLKLKLHV------------- 775
XP_001843671.1[Culex         VYSKAGHGMGKPTAKKIEESTDILTFIYKTLKLKLTF------------- 738
ACI03586.2[Eurygaster        VETKAGHGAGKPTNKRIEEQVDILCFLMNSMNLKFIE------------- 711
EFX79244.1[Daphnia           IETKAGHGANKPTSKIIDEHSDVFAFLARALNLEFQS------------- 708
NP_001004050.1[Sus           VDTKAGHGAGKPTAKVIEEVSDMFAFIARCLNIDWIP------------- 710
```

Figure S3. *Cont.*

```
XP_006061999.1[Bubalus       VDTKAGHGAGKPTAKVIEEVSDMFAFIARCLNIDWIQ------------- 710
NP_001244843.1[Macaca        VDTKAGHGAGKPTAKVIEEVSDMFAFIARCLNIDWI-------------- 709
NP_112614.1[Rattus           VDTKAGHGPGKPTAKVIEEVSDMFAFIARCLNIEWIQ------------- 710
XP_005368679.1[Microtus      VDTKAGHGAGKPTAKVIEEVSDMFAFIARCLNIEWIQ------------- 710
NP_035286.1[Mus              VDTKAGHGAGKPTAKVIEEVSDMFAFIARCLNIEWIQ------------- 710
XP_005154718.1[Melopsittacus VDTKAGHGAGKPTAKVIEEVSDMFAFIARCLNLDWIE------------- 715
XP_005518296.1[Pseudopodoces VDTKAGHGAGKPTAKVIEEVSDMFAFIARCLNLEWIE------------- 715
NP_001006410.1[Gallus        VDTKAGHGAGKPTAKVIEEVSDMFAFIARCLNLDWIE------------- 710
XP_009046664.1[Lottia        IDTKSGHGAGKPTKKVIEEVTDIYSFLYTTLNLEW--------------- 707
XP_005095067.1[Aplysia       VDTKSGHGFGKPTAKVIEEISDIYSFIYQTIGLEW--------------- 705
EFN66352.1[Camponotus        IDTKAGHGGGKPTMKVLEESTDILSFIVQSLGLEFKS------------- 706
EZA56288.1[Cerapachys        IDTKAGHGGGKPTMKVIEESTDILSFIVQSLGLEF--------------- 704
EFN76622.1[Harpegnathos      IDTKAGHGSGKPTMKVIEESTDILSFIVQSLGLVFKS------------- 764
XP_003394688.1[Bombus        IETKAGHGGGKPTMKVIEESTDILAFIVKSLNLEFKL------------- 763
XP_395364.2[Apis             IETKAGHGGGKPTMKVIEESTDILAFIVKSLDLEF--------------- 705
XP_001603578.2[Nasonia       IDVKAGHGRGKPTSKVIDESTDILSFVVQTLNLEF--------------- 703
XP_008556849.1[Microplitis   IDTKAGHGRGKPTTKVIEETRDILVFIIKTLNLKF--------------- 709
KDR21572.1[Zootermopsis      IETKAGHGLGKPTAKLVSNYLNVCSF------------------------ 701
ERL94303.1[Dendroctonus      IETKAGHGAGKPTAKMIEEMTEIFCFISKALGLPFSE------------- 703
XP_008193477.1[Tribolium     IETRAGHGAGKPTSKIIEEVTDTFCFISRALNLTF--------------- 706
AAA24925.1[Elizabethkingia   IETNAGHGAGRSTEQVVMENADLLSFALYEMGIKNLK------------- 705
pdb|1YR2|[Sphingomonas       IETRAGHGSGKPIDKQIEETADVQAFLAHFTGLTPRPWSSVDKLAAALEH 736
AAD31004.1[Myxococcus        IEANAGHGGADQVAKAIESSVDLYSFLFQVLDVQG----AQGGVAAQGR- 689
                             :  .:***        : .  :   *
```

Figure S4. Phylogenetic tree of Clustal W alignment *of Eurygaster integriceps* PEP. *Eurygaster integriceps* protein ID is boxed for clarification. The program phylogeny.fr [1] was used to generate the tree using the Clustal W output. PEP Isoforms obtained from the BLASTX were removed from the analysis.

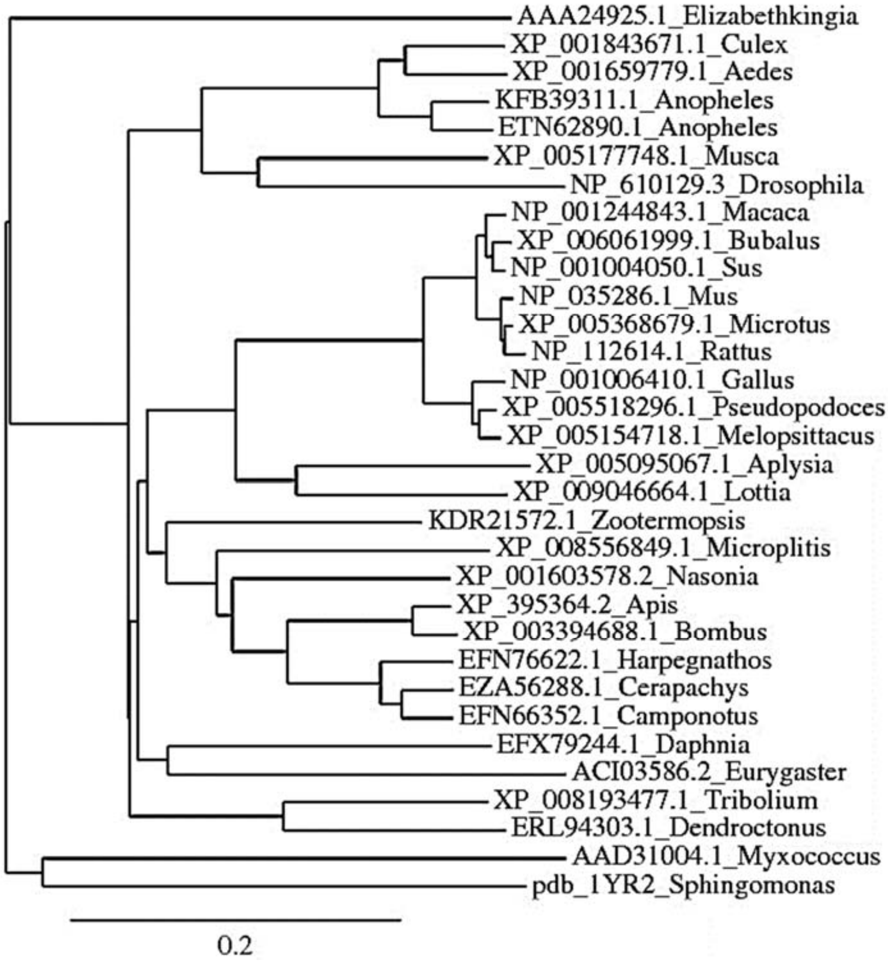

Table S1. Transcription Factor binding sites identified in the 5'UTR using TFSearch.

TF Matrix No.	ID	Location (5' bp No.)	Function Identified in Drosophila
M00012	CF2-II	185	Late activator in follicle cells
M00019	Dfd	35, 290	Deformed
M00022	Hb	169, 170, 171, 172, 173, 301	Hunchback
M00028	HSF	55,176, 178	Heat shock factor
M00091	BR-C Z1	28, 288	
M00093	BR-C Z3	50	Ecdysone-responsive key regulator of metamorphosis during 3rd instar and early pupal development.
M00094	BR-C Z4	292, 299	
M00199	AP-1	317	
M00266	Croc	289, 299	Required for normal head development

Table S2. BLAST results for the Eurygaster integriceps prolyl endoprotease compared to the following databases and individually when designated with the following symbols: eukaryotes (taxid:2759), insects (taxid:6960), bacteria (taxid:2), fungi (taxid:4751), archaea (taxid:2157), yeast (taxid:4932). PEP Isoforms obtained from the BLAST were removed from the analysis.

Accession	Organism	Query Coverage	Identity
ACI03586.2	*Eurygaster integriceps*	100%	100%
ERL94303.1	*Dendroctonus ponderosae*	75%	52%
XP_008193477	*Tribolium castaneum*	75%	50%
EFX79244.1	*Daphnia pulex*	75%	56%
NP_001004050.1	*Sus scrofa*	75%	50%
XP_006061999	*Bubalus bubalis*	75%	50%
NP_001244843	*Macaca mulatta*	75%	50%
NP_112614.1	*Rattus norvegicus*	75%	50%
XP_005368679	*Microtus ochrogaster*	75%	50%
NP_035286.1	*Mus musculus*	75%	50%
XP_005154718	*Melopsittacus undulates*	76%	50%
XP_005518296	*Pseudopodoces humilis*	76%	50%
NP_001006410	*Gallus gallus*	75%	50%
XP_009046664	*Lottia gigantean*	75%	52%
XP_005095067	*Aplysia californica*	75%	51%
EFN66352.1	*Camponotus floridanus*	75%	53%
EZA56288.1	*Cerapachys biroi*	75%	53%
EFN76622.1	*Harpegnathos saltator*	75%	53%
XP_003394688	*Bombus terrestris*	76%	55%
XP_395364.2	*Apis Mellifera*	75%	55%
XP_001603578	*Nasonia vitripennis*	75%	55%
XP_008556849	*Microplitis demolitor*	75%	51%
KDR21572.1	*Zootermopsis mevadensis*	74%	55%
ETN62890.1	*Anapholes darling*	75%	51%
KFB39311.1	*Anapholes sinensis*	75%	49%

Table S2. *Cont.*

Accession	*Organism*	Query Coverage	Identity
XP_005177748	*Musca domestica*	74%	51%
NP_610129.3	*Drosophila melanogaster*	76%	51%
AAA24925	*Elizabethkigia meningoseptica*	99%	35%
pdb|1YR2	*Sphingomonas capsulata*	97%	39%
AAD31004.1	*Myxococcus xanthus*	99%	33%

Reference

1. Dereeper, A.; Guignon, V.; Blanc, G.; Audic, S.; Buffet, S.; Chevenet, F.; Dufayard, J.F.; Guindon, S.; Lefort, V.; Lescot, M.; *et al. Phylogeny.fr: Robust Phylogenetic Analysis for the Non-Specialist*. *Nucleic Acids Res.* **2008**, *36*, W465–W469.
